# Characterising a New Cannabis Trend: Extensive Analysis of Semi‐Synthetic Cannabinoid‐Containing Seizures From Germany

**DOI:** 10.1002/dta.3886

**Published:** 2025-03-19

**Authors:** Marica Hundertmark, Laura Besch, Jörg Röhrich, Tanja Germerott, Cora Wunder

**Affiliations:** ^1^ Department of Forensic Toxicology, Institute of Legal Medicine University Medical Center Mainz Mainz Germany; ^2^ Dez. 33 Chemistry/Toxicology, State Office of Criminal Investigation Rhineland‐Palatinate Forensic Science Institute Mainz Germany

**Keywords:** CBD‐derived cannabinoids, CBG‐chemotype, hemp‐compliant products, hexahydrocannabinol, tetrahydrocannabinol

## Abstract

In May 2022, semi‐synthetic cannabinoids (SSC) appeared on the European drug market, claiming to offer a legal alternative with cannabimimetic effects. Since then, the use of hexahydrocannabinol (HHC) has quickly become very popular and derivatives, among them heptyl‐analogs with prolonged alkyl‐sidechains and acetylated forms, have appeared. First HHC‐bans were introduced in some EU countries in early 2023. As only limited data is available on this dynamic consumption trend, this study aims to analyse a seizure collective comprehensively.

A liquid chromatography–tandem mass spectrometry method was validated for quantification of (R,S)‐HHC, Δ^8^‐THC, Δ^9^‐THC, CBD, CBG, CBN, (R,S)‐HHC‐O and CBN‐O and applied to a collective of 80 SSC‐containing products including flowers, resins, edibles, vape liquids and papers. Further derivatives, among them (R,S)‐HHCP, Δ^9^‐THCP, Δ^8^‐THCP, (R,S)‐HHCP‐O, (R,S)‐H4CBD, THC‐O and THCP‐O were qualitatively evaluated.

HHC‐content was characterised by extreme fluctuations from <LOQ up to 70.9 wt‐%. Δ^9^‐THC was detected in most seizures, with around a quarter of samples exceeding the EU‐legal limit for hemp (< 0.3 wt‐% Δ^9^‐THC). Δ^8^‐THC was rarely found in elevated levels which might indicate residues of HHC‐synthesis. H4CBD was the most frequently detected SSC‐derivative, followed by heptyl‐analogs. Acetates played a minor role and were usually only detected in traces, while elevated levels occurred rarely. Unusual cannabinoid compositions were detected in cannabis carrier material, including extreme CBD‐concentrations (up to 67.3 wt‐%) and CBG‐dominant cultivars.

Systematic investigation of seizures provides information for assessing the risk to consumers and is a valuable basis for the interpretation of findings in biological material.

## Graphical abstract

A LC–MS/MS method has been validated for analysis of semi‐synthetic cannabinoids (SSC) and selected phytocannabinoids in seizures. Further SSC‐derivatives were implemented into the method for qualitative detection. The methodology was applied to a sample collective (*n* = 80) including cannabis flowers, resins, edibles and vape liquids. Results indicate high fluctuations in HHC‐contents and complex mixtures of SSC‐derivatives in the products. Unusual cannabinoid compositions were observed in some herbal carrier materials such as artificially enhanced CBD‐contents and CBG‐dominant material.

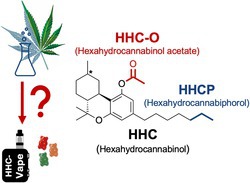

## 1 Introduction

After decades of prohibition, regulations surrounding the plant *Cannabis sativa* are being reconsidered, leading to fundamental transformations in legislation worldwide. Debates on the legalisation of ‘*marijuana*’ for medical [1, 2] or recreational [3] purposes dominate the media in many countries. These laws are primarily defined by the main intoxicating cannabinoid of *‘marijuana’*, Δ^9^‐tetrahydrocannabinol (Δ^9^‐THC). In addition to *‘drug‐type’* cannabis, however, there is also a chemotype with low Δ^9^‐THC and high cannabidiol (CBD) content, known as *‘fibre‐type’*. Since around 2016, the marketing of a diverse range of CBD‐infused products has become a widespread putatively health‐beneficial ‘lifestyle’ trend [3, 4]. Also, the cultivation of CBD‐dominant ‘*hemp*’ is currently subject to legal changes. In particular, the US Agriculture Improvement Act of 2018 (‘Farm Bill 2018’) has led to complex consequences on the CBD‐market [5]. While ‘*hemp*’ was removed from the definition of ‘*marijuana*’ in the Controlled Substances Act, *C. sativa* or products of the plant with a Δ^9^‐THC content of ≤ 0.3% by dry weight were declared legal [5, 6]. Immediately, an overproduction of CBD resulted, which, in combination with legal loopholes, led to it being used as a precursor for ‘CBD‐derived cannabinoids’. In contrast to CBD, most of these cannabinoids, also known as semi‐synthetic cannabinoids (SSC), are characterised by psychoactive effects due to their cannabinoid receptor 1 (CB_1_) agonism [7]. A wide variety of ‘hemp‐compliant’ products enriched with SSCs have quickly become available. In particular, herbal preparations sprayed with hexahydrocannabinol (HHC) such as low‐THC cannabis flowers or resin, e‐cigarette liquids and a large selection of sweets, like gummies, brownies and biscuits, are popular forms of consumption [6, 8].

**FIGURE 1:**
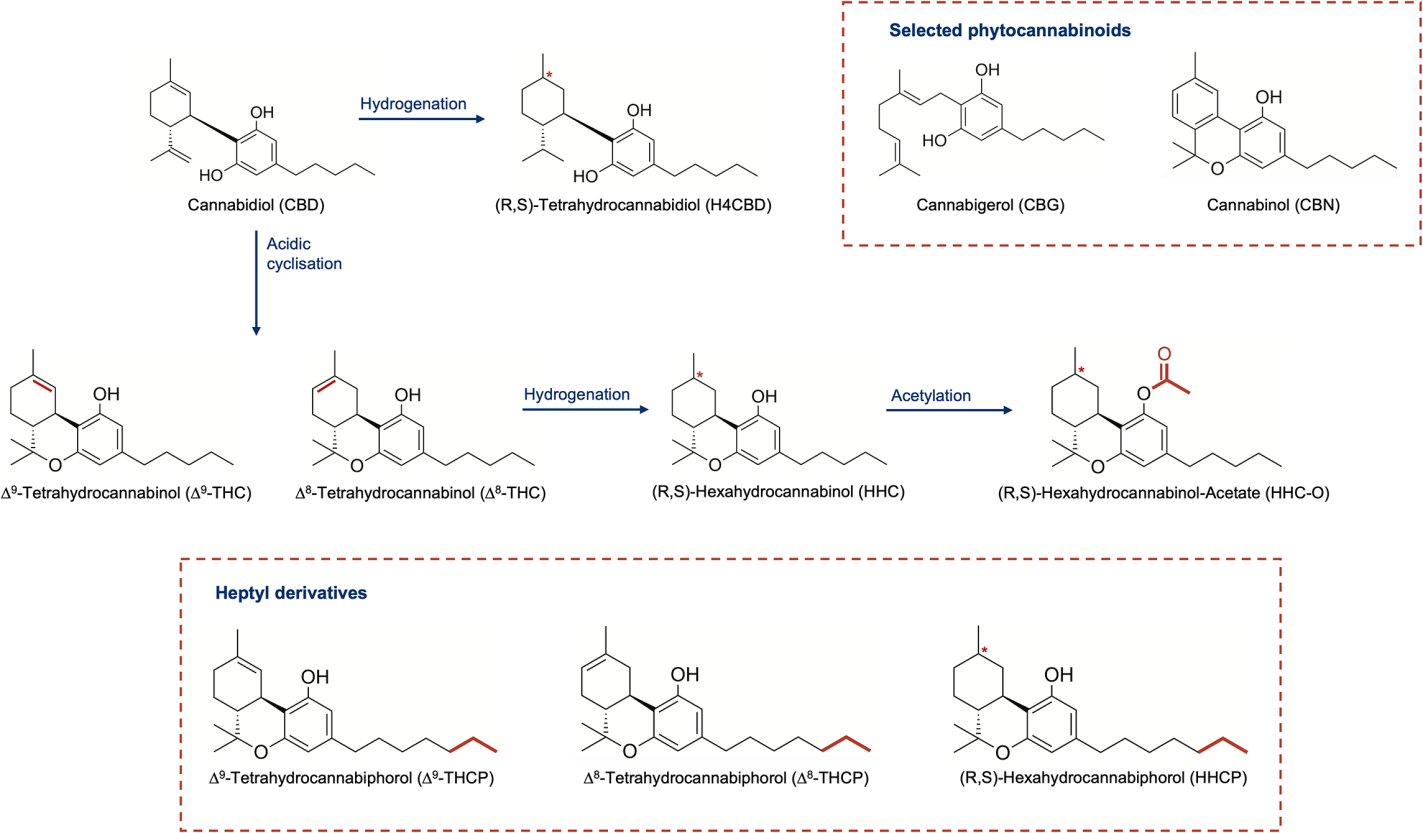
Synthesis and structural formula of HHC as well as structural formulas of selected SSC‐derivatives and selected phytocannabinoids included in the analysis.

The first SSC observed in the US around September 2019 was Δ^8^‐tetrahydrocannabinol (Δ^8^‐THC, Figure 1) [8], a positional isomer of Δ^9^‐THC differing only in the location of the double bond in the cyclohexyl ring. In a broader interpretation of the US ‘Farm Bill’ definitions, Δ^8^‐THC can be considered as legal at federal level and has spread rapidly in many states [7]. However, since Δ^8^‐THC is listed in Annex II of the 1971 United Nations Convention on Psychotropic Substances [9], it is prohibited by national laws in most countries and has not yet gained international popularity. Around September 2021, HHC was identified as second SSC in the USA. After acidic cyclisation of CBD, the double bond in the cyclohexyl ring of the resulting mixture of Δ^8^‐ and Δ^9^‐THC can be hydrogenated to form HHC. As this reaction forms another chiral centre, two possible diastereomers may be formed, referred to as (R)‐ and (S)‐HHC [10, 11]. Unlike Δ^8^‐THC, HHC was not legally regulated at that time and spread globally quickly. In Europe, HHC was first observed in Denmark in May 2022. Since 21^st^ October 2022, it has been monitored by the EU Early Warning System as a novel psychoactive substance (NPS), as it had already reached 70% of EU Member States up to December 2022 [8, 12]. Following a successful market entry of HHC in Europe, first structurally modified derivatives were soon reported to the European Union Drug Agency (EUDA). An acetylated version of HHC, also referred to as HHC‐O was observed in August 2022 by Hungarian authorities. Hexahydrocannabiphorol (HHCP), an HHC‐homolog with a prolonged heptyl‐sidechain, was first observed in November 2022 by Slovenia [8], followed by tetrahydrocannabidiol (H4CBD), a saturated version of CBD, in December 2022. Similar to HHCP, Δ^8^‐ resp. Δ^9^‐tetrahydrocannabiphorol (Δ^8^‐THCP, Δ^9^‐THCP) appeared in March 2023 [13]. Acetates of heptyl derivatives, HHCP‐O, THCP‐O, have been observed for the first time in Europe in September 2023 [14, 15].

These extremely dynamic developments led to the first national bans on HHC and its derivatives in some EU countries beginning in 2023 [16, 17]. At the same time, there is also a need for further analytical developments in forensic laboratories. HHC was already described in literature in the 1940s [18], but it has received systematic research interest only recently. Publications on analytical method development in seizures [19] and biological material [20], preliminary pharmacokinetic [21, 22, 23, 24] and pharmacodynamic data [25, 26] have become available. At present, current publications often only provide qualitative data or examine isolated seizures [10, 27]. The aim of this work was therefore to provide HHC‐contents of a larger seizure collective. Since also only limited data is available on derivatives beyond HHC so far, HHC‐O was also quantified, and further derivatives were analysed qualitatively. Hence, in a two‐step process, further derivatives were first identified in seizures by mass spectrometric experiments and then, the determined mass transitions were applied to the collective. To draw comprehensive conclusions about the possible synthesis residues and the herbal cannabis material to which SSCs were applied, Δ^8^‐THC, Δ^9^‐THC, CBD, cannabigerol (CBG) and cannabinol (CBN) were also quantified.

## 2 Material and Methods

### 2.1 Material

(R)‐HHC, (S)‐HHC and CBG‐d9 were obtained from Cayman chemicals (Ann Arbor, Michigan, USA). Δ^9^‐THC, CBD, CBN, 11‐nor‐9‐carboxy‐Δ^9^‐tetrahydrocannabinol‐d9 (Δ^9^‐THC‐COOH‐d9) and CBD‐d9 were purchased from LGC (Wesel, Germany). Δ^9^‐tetrahydrocannabinolic acid A (Δ^9^‐THCAA) was obtained from THC Pharm (Frankfurt a. M., Germany). Δ^8^‐THC, CBG, tetrahydrocannabivarin (THCV), cannabidivarin (CBDV), cannabicyclol (CBL), cannabichromene (CBC), Δ^9^‐THC‐d3, CBN‐d3 and the further acidic cannabinoids, cannabigerolic acid (CBGA), cannabinolic acid (CBNA), cannabidiolic acid (CBDA), tetrahydrocannabivarinic acid (THCVA), cannabidivarinic acid (CBDVA), cannabicyclolic acid (CBLA) and cannabichromenic acid (CBCA), were from Cerilliant (Round Rock, Texas, USA). (R)‐ und (S)‐HHCP at a concentration of approx. 0.9 mg/mL as well as (S)‐HHCP‐O at a concentration of 1 mg/mL was kindly provided by the Institute of Legal Medicine in Düsseldorf. Methanol (LC–MS‐grade) was from Honeywell (Seelze, Germany). Water (LC–MS‐grade), acetonitrile (LC–MS‐grade) were purchased from Carl Roth (Karlsruhe, Germany). Ammonium acetate (eluent additive for LC–MS) was bought from Sigma‐Aldrich (St. Louis, Missouri, USA).

Acetate derivatives (Δ^8^‐THC‐O, Δ^9^‐THC‐O, (R)‐HHC‐O, (S)‐HHC‐O, CBN‐O) were synthesised based on their deacetylated precursors according to Holt et al. [28]. Briefly, an amount of 10 μg of reference substance, previously evaporated to dryness, was dissolved in 50 μL pyridine and 25 μL acetic anhydride and incubated in closed vessels on a heat block (75°C) overnight.

### 2.2 Seizure Collective

Analysis covered 79 SSC‐containing samples from 20 seizures, among them cannabis flowers, resin, edibles (gummies), vape‐liquids and papers (presumably soaked with vape liquids). Samples were confiscated between December 2021 and December 2023 by the State Office of Criminal Investigation Rhineland‐Palatinate. A detailed description of seized material is given in Table S1. Moreover, H4CBD containing gummies were bought in a local shop in Mainz in September 2023. Aliquots of flowers, resin and gummies were weighted (approx. 10–300 mg) and sonicated in methanol (1–10 mL) at 60°C for 30 min. Extracts were stored at −20°C until analysis.

### 2.3 Quantitative Analysis

#### 2.3.1 LC–MS/MS Instrumentation and Analytical Parameters

Analysis was performed using a LC–MS/MS system from Agilent (Waldbronn, Germany). Chromatography was conducted using a 1290 Infinity II LC system, coupled via Jet Stream interface (ESI) to a 6495C triple quadrupole mass spectrometer. The system contained an Agilent Infinity Lab Poroshell 120 EC‐C18 column (2.1 × 150 mm/2.7 μm) guarded by an Infinity Lab Poroshell 120 EC‐C18 column (2.1 × 5 mm/2.7 μm). The mobile phase consisted of (A) 10 mM ammonium acetate in water and (B) methanol. The column temperature was 50°C, injection volume was 5 μL and flow rate was 0.5 mL/min. A step gradient elution was carried out starting at 60% B for 2 min and then gradually increased to 77% B at 8 min. These conditions were kept for 4 min before B was increased to 95% for column washing for 2 min, followed by a re‐equilibration period of 3 min (total run time 20 min). For mass‐selective detection, electrospray parameters were as follows: gas flow 15 L/min at 250°C; nebulizer 20 psi, sheath gas flow 12 L/min at 400°C; capillary voltage ± 4000 V. Analytes were detected in multiple reaction monitoring (MRM) mode using the transitions shown in Table 1. An internal standard (ISTD) solution containing 100 ng/mL Δ^9^‐THC‐d3, 5 ng/mL CBD‐d9, 10 ng/mL CBG‐d9, 1 ng/mL CBN‐d3 and 4 ng/mL Δ^9^‐THC‐COOH‐d9 was prepared in methanol. The assignment of analytes to deuterated ISTD is shown in Table 1.

**TABLE 1:** Calibration scheme, QC levels and mass transitions for quantitative MRM‐analysis of seizures.

	K1	K2	K3	K4	K5	K6	K7	K8	K9	QC_low_	QC_med_	QC_high_	Precursor	Target	Qualifier	ISTD
Neutral cannabinoids																
(R)‐HHC, (S)‐HHC	1	5	10	25	50	100	200	400	500	2.5	12.5	250	+317	193.0 (26 eV)	137.0 (22 eV)	THC‐d3
Δ^8^‐THC, Δ^9^‐THC	1	5	10	25	50	100	200	400	500	2.5	12.5	250	+315.2	193.1 (22 eV)	123.0 (40 eV)	THC‐d3
CBD	1	5	10	25	50	100	200	400	500	2.5	12.5	250	+315.2	193.1 (22 eV)	123.0 (40 eV)	CBD‐d9
CBG	1	5	10	25	50	100	200	400	500	2.5	12.5	250	+317.0	193.0 (26 eV)	123.0 (40 eV)	CBG‐d9
CBN	1	5	10	25	50								−309.2	279.1 (40 eV)	221.1 (52 eV)	CBN‐d3
Cannabinoid acids																
Δ^9^‐THCAA	1	5	10	25	50	100				2.5	12.5		−357.2	242.2 (34 eV)	191.1 (48 eV)	Δ^9^‐THC‐COOH‐d9
CBDA	1	5	10	25	50	100	200	400	500	2.5	12.5	250	−357.2	339.2 (22 eV)	245.2 (30 eV)	Δ^9^‐THC‐COOH‐d9
CBGA	1	5	10	25	50	100	200	400	500	2.5	12.5	250	−359.2	341.2 (18 eV)	191.1 (40 eV)	Δ^9^‐THC‐COOH‐d9
CBNA	1	5	10	25	50					2.5	12.5		−353.2	309.2 (26 eV)	279.1 (38 eV)	Δ^9^‐THC‐COOH‐d9
Acetylated cannabinoids																
(R)‐HHC‐O		0.5	1	5	10	25	50			0.75	12.5	75	+359.2	317.0 (18 eV)	193.0 (40 eV)	THC‐d3
(S)‐HHC‐O		0.5	1	5	10	25	50	100		0.75	12.5	75	+359.2	317.0 (18 eV)	193.0 (40 eV)	THC‐d3
CBN‐O	0.25	0.5	1	5	10	25	50			0.75	12.5	75	+353.2	311.0 (18 eV)	223.0 (30 eV)	CBN‐d3

*Note:* ISTD‐Solution: Δ^9^‐THC‐d3 (+318.3 ➔ 196.1 (22 eV)), CBD‐d9 (+324 ➔ 202 (22 eV)), CBG‐d9 (+326.2 ➔ 202 (26 eV)), CBN‐d3 (−312 ➔ 282 (40 eV), Δ^9^‐THC‐COOH‐d9 (−352.3 ➔ 308.3, 22 eV).

#### 2.3.2 Validation

Validation was performed in accordance with the guidelines of the German Society of Toxicological and Forensic Chemistry (GTFCh) [29, 30] and included the parameters selectivity, linearity of calibration, analytical limits, accuracy (bias), intra‐ and inter‐day precision and matrix effects. Data was statistically evaluated using Valistat 2.00.1 software (Arvecon; Walldorf, Germany). Method selectivity was determined using methanol as blank matrix. Interferences of mass transitions of analytes by the corresponding deuterated standards were checked by direct measurement of the ISTD‐solution. Moreover, further minor cannabinoids among them THCV, THCVA, CBDV, CBDVA, CBL, CBLA, CBC and CBCA were tested for potential interferences in the selected mass transitions. Three separate calibration sets were prepared for neutral cannabinoids, cannabinoid acids and acetylated cannabinoids. Linearity of calibration was tested by a four‐fold measurement of the calibration series covering at least five calibrators [30]. Calibration ranges for each analyte are given in Table 1. Analytical limits were determined in accordance with the DIN 32645 [31]. The DIN 32645 is a document that defines the terms ‘limit of detection’ and ‘limit of quantification’ of analytical methods and describes how these can be estimated using appropriate methodological procedures and calculation formulae. According to the calibration curve method, six evenly distributed calibrators in the range of the expected detection limit (neutral cannabinoids and cannabinoid acids: 0.25–1.5 ng/mL, acetylated cannabinoids: 0.125–0.75 ng/mL) were prepared. Accuracy (bias), intra‐ and inter‐day precision were evaluated by analysing quality control (QC) samples at low, medium and high concentrations relative to the calibration range (Table 1) in duplicate on eight different days. Acceptance criteria was a bias of ± 15% (± 20% near the limit of quantification) and a relative standard deviation (RSD) of ≤ 15% (≤ 20% near the limit of quantification) for intra‐ and inter‐day precision. Matrix effects were tested using the standard addition method exemplarily for each sample type including cannabis flowers, resin and gummies. In accordance with the recommendations by Hasegawa et al. [32] six samples in appropriate dilutions were prepared and spiked with ISTD‐solution. Five of them were spiked with increasing concentrations of standard (10 μL in methanol:acetonitrile:water (3/3/2, v/v/v), 30 ng/mL steps for (R)‐HHC, (S)‐HHC, Δ^8^‐THC, Δ^9^‐THC, CBD, CBDA, CBG and CBGA, 3 ng/mL steps for CBN, CBNA, THCAA, CBN‐O, (R)‐HHC‐O, (S)‐HHC‐O), ensuring that the calibration ranges were not exceeded. One sample was spiked with 10 μL solvent (methanol:acetonitrile:water (3/3/2, v/v/v)). Quantification via standard addition was verified by plotting either the absolute peak area or the relative peak area (including the ISTD) against the spiked concentration. The resulting correlation coefficients should be > 0.99 [32]. Quantitative results determined by standard addition, similar to acceptance criteria for QC samples [33], should not deviate by more than ± 30% from the previously determined quantifications using the calibration model.

### 2.4 Qualitative Analysis of SSC‐Derivatives

For initial identification of SSC‐derivatives in (diluted) extracts, mass‐spectrometric experiments were carried out on an UHPLC 1290 Infinity coupled to a 6490 triple quadrupole mass spectrometer from Agilent (Waldbronn, Germany). After precursor ions of potential derivatives were identified by MS2‐Scan‐mode, targeted product ion scans were carried out in positive and negative mode at different collision energies (range 13–50 eV) for determination of suitable mass transitions for subsequent qualitative MRM‐detection. The underlying chromatography was based on the in‐house THC‐routine analysis and performed on a C18‐column (ZORBAX Eclipse Plus C18 Rapid Resolution HD 2.1 × 100 mm 1.8 μm, Agilent). For each compound detected two suitable mass transitions were chosen and implemented qualitatively in the chromatographic conditions to be validated (according to Section 2.3.1.).

### 2.5 Sample Analysis

For quantitative analysis of seizure extracts, 1:1,000 dilutions in methanol:acetonitrile:water (3/3/2, v/v/v) were measured and spiked with an equal amount of ISTD‐solution (20 μL each). If an analyte was out of calibration range, further dilutions (1:100, 1:10,000, 1:100,000, 1:1,000,000) were subsequently measured. The concentrations determined in the extracts were used to determine weight percentages (wt‐%) according to the initial weighing of the samples. For cannabinoids of which neutral and acidic forms were measured, including Δ^9^‐THC, CBD, CBG and CBN, a total value corresponding to the neutral cannabinoid was calculated (total cannabinoid content = amount [neutral form] + amount [acidic form] * molar mass [neutral form] / molar mass [acidic form]) [34]. Qualitative analysis was performed in either 1:1,000 or 1:10,000 dilutions and response ratios in relation to Δ^9^‐THC‐d3 were determined. Criteria for a positive identification were appropriate retention time, detection of two mass transitions (as shown in Section 3.2.) with adequate signal intensity (S/*N* ≥ 3) and a consistent target/qualifier‐ratio. For better comparability of the values, the solvent volume was eliminated by multiplication and sample weight by division. Data evaluation was done using Agilent MassHunter Workstation Software (Version 10.1, ©2019), Microsoft Excel 365 (Version 16.83, ©2024) and SPSS (Version 23.0.0.3, ©2016).

## 3 Results

### 3.1 Quantitative Analysis: Validation Results

**FIGURE 2:**
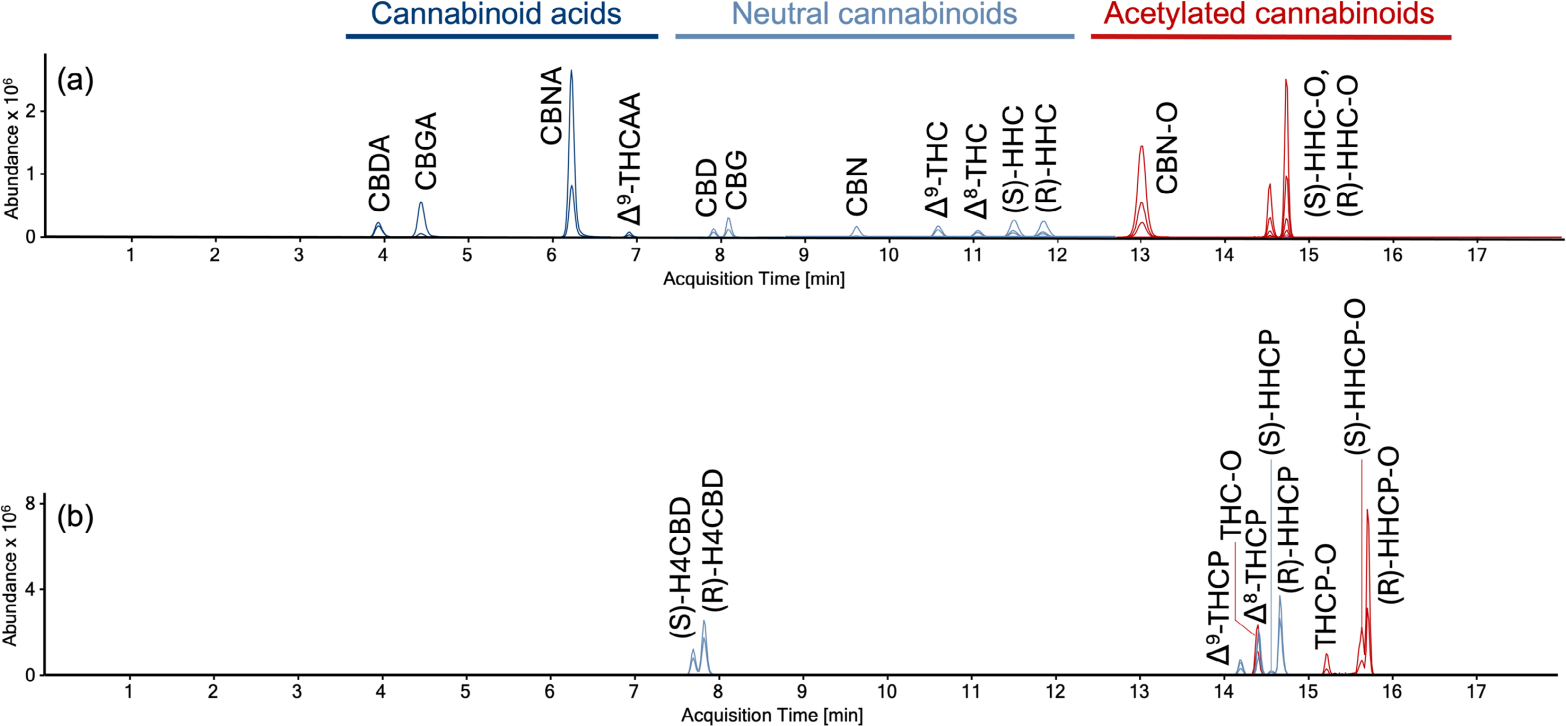
MRM‐Chromatogram of a standard mixture containing all quantified substances in seizures at equal concentrations (4.2 ng/mL each, (a)). MRM‐Chromatogram as an overlay of several seizures covering all SSC‐derivatives monitored qualitatively (b).

**FIGURE 3:**
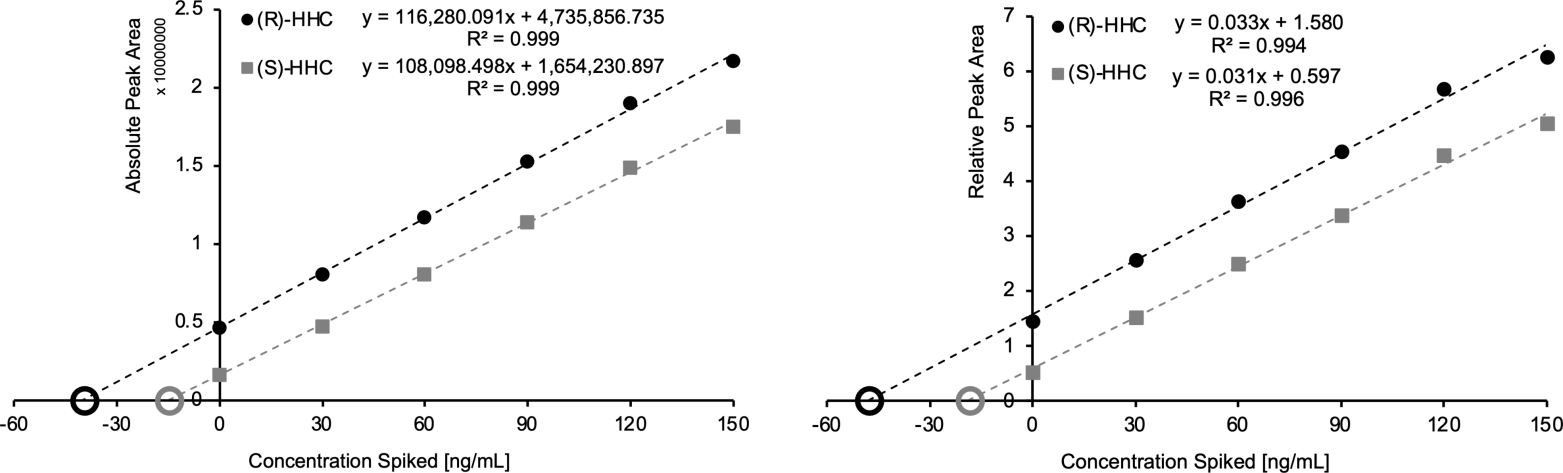
Evaluation of standard addition data sets for (R)‐ and (S)‐HHC in a cannabis flower (No. 2, Table S2). Analysis was performed in 1:10,000 dilution in methanol:acetonitrile:water (3:3:2, v/v/v). Absolute (left) and relative peak areas (right, Δ^9^‐THC‐COOH‐d9 as internal standard) are plotted against the concentrations spiked (+30 ng/mL steps). Quantification for (R)‐HHC resulted in 53.4 ng/mL using the validated method, 40.7 ng/mL (−23.7%) by absolute quantification and 48.3 ng/mL (−9.43%) by relative quantification using the standard addition approach. Quantification for (S)‐HHC resulted in 18.1 ng/mL using the validated method, 15.3 ng/mL (−15.4%) by absolute quantification and 19.3 ng/mL (6.93%) by relative quantification using the standard addition approach.

An overview of the chromatographic separation is shown in Figure 2a. No interference was observed for methanol and all cannabinoids tested, except CBL which showed co‐elution with Δ^8^‐THC. However, target/qualifier‐ratios differed clearly between Δ^8^‐THC (+315.2 ➔ 193.1 > 123.0) and CBL (+315.2 ➔ 123.0 > 193.1). A linear calibration model without weighting could be used for all analytes (Variance homogeneity tested by a Cochran test across all concentrations, significance 99%; Mandel linearity test, significance: 99%) with a correlation coefficient of > 0.99. Analytical limits were consistently below the lowest calibrator (results see Table 2). Accuracy (bias), intra‐ and inter‐day precision were within the acceptance criteria for all analytes (see Table 2). Moreover, all quantified values via standard addition were within the accepted ± 30% limit compared to the previously determined quantification using the calibration model to be validated. Linear regressions in standard addition experiments showed consistently correlation coefficients > 0.99. Exemplarily, the evaluation of a standard addition set for (R)‐ and (S)‐HHC in a cannabis flower is shown in Figure 3. All further results for standard addition are listed in Table S2.

**TABLE 2:** Validation results for quantitative analysis of seizures. The table shows results for limit of detection (LOD), limit of quantification (LOQ) as well as accuracy, intra‐day‐ and inter‐day precision at low, medium (med) and high QC level.

Analyte	Analytical limits ng/mL		Accuracy (bias) % (n = 8)			Intra‐day precision % (*n* = 8)			Inter‐day precision % (n = 8)		
	LOD	LOQ	low	med	high	low	med	high	low	med	high
Neutral cannabinoids											
(R)‐HHC	0.18	0.54	−3.1	−9.1	2.1	3.5	4.1	2.8	8.3	5.4	5.7
(S)‐HHC	0.13	0.41	−6.3	−9.5	3.5	4.6	3.5	2.6	8.9	5.3	5.5
Δ^8^‐THC	0.16	0.48	−6.9	−7.9	1.3	3.9	4.0	4.1	6.2	5.2	6.3
Δ^9^‐THC	0.10	0.33	−6.6	−7.0	0.49	3.4	3.8	2.2	6.8	4.5	3.8
CBD	0.18	0.55	−4.2	−9.4	−1.1	4.4	3.5	4.0	7.0	5.4	6.7
CBG	0.12	0.39	−3.9	−6.7	4.9	4.6	4.0	3.2	7.3	5.2	5.6
CBN	0.19	0.57	2.9	4.1	na	3.8	4.0	na	9.1	6.9	na
Cannabinoid acids											
THCAA	0.23	0.69	8.8	5.3	na	3.9	4.2	na	6.4	7.2	na
CBDA	0.028	0.10	−1.6	4.0	1.3	8.2	6.2	7.8	13.4	6.8	7.8
CBGA	0.14	0.44	3.5	5.3	2.1	8.7	4.4	6.5	9.7	4.4	7.3
CBNA	0.049	0.17	11.3	6.1	na	5.9	5.9	na	6.2	5.9	na
Acetylated cannabinoids											
(R)‐HHC‐O	0.084	0.26	−11.6	−2.4	na	6.2	6.4	na	6.2	2.4	na
(S)‐HHC‐O	0.16	0.46	−9.1	−3.0	−5.0	4.4	8.8	4.2	8.2	9.0	8.6
CBN‐O	0.052	0.17	−5.5	−1.1	na	4.7	3.0	na	11.9	10.4	na

### 3.2 Qualitative Analysis: Identification and Implementation of SSC‐Derivatives

Using MS2 scans, (R)‐ and (S)‐HHCP, Δ^8^‐ and Δ^9^‐THCP, (R)‐ and (S)‐H4CBD, (R)‐ and (S)‐HHCP‐O could be identified in seizures and comprehensive mass spectra were recorded by product ion scans (Figure S1). Since none of the initially analysed seizures contained Δ^8^‐ and Δ^9^‐THCP‐O, a THCP‐positive sample (No.28, Table S4) was acetylated according to Holt et al. (2022) [28]. Also Δ^8^‐ and Δ^9^‐THC‐O, derived from the respective reference substances, were monitored qualitatively. Suitable mass transitions for these analytes were determined (Table 3) and implemented qualitatively in the validated chromatographic conditions (Figure 2b).

**TABLE 3:** Mass transitions for qualitative MRM‐analysis in seizures. For each analyte two mass transitions with corresponding collision energies (ce) are given.

Analyte	Precursor ion	Mass transition 1 product ion, ce [eV]	Mass transition 2 product ion, ce [eV]
(R)‐ and (S)‐HHCP	+345.0	221.0, 38	137.0, 34
Δ^8^‐ and Δ^9^‐THCP	+343.0	221.0, 30	287.0, 15
(R)‐ and (S)‐H4CBD	+319.0	83.0, 22	181.0, 22
(R)‐ and (S)‐HHCP‐O	+387.0	345.0, 18	221.0, 40
Δ^8^‐ and Δ^9^‐THCP‐O	+385.0	343.0, 22	221.0, 30
Δ^8^‐ and Δ^9^‐THC‐O	+357.2	315.0, 22	259.0, 25

**FIGURE 4:**
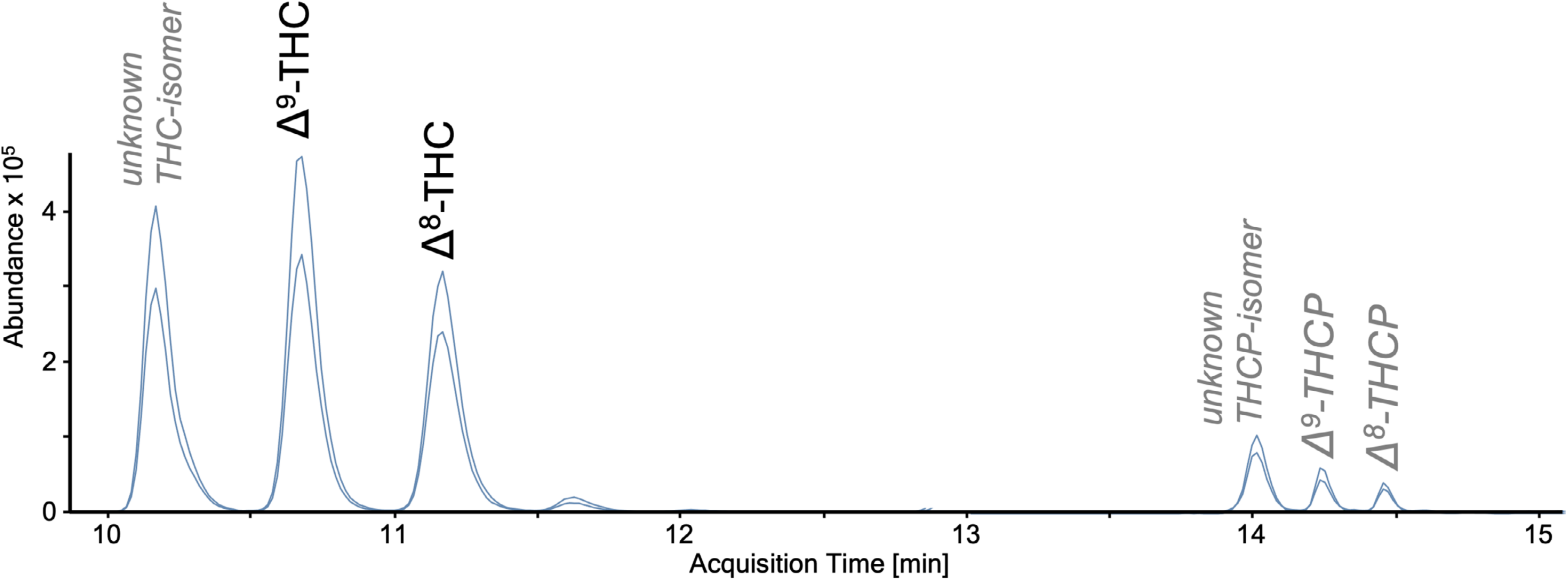
MRM‐Chromatogram of sample No. 2 showing the occurrence of THC‐ resp. THCP‐isomers, the definite assignment Δ^9^‐THC and Δ^8^‐THC based on reference substances (labels in black) as well as the putative assignment of Δ^9^‐THCP and Δ^8^‐THCP (labels in grey and italic).

It is assumed that heptyl‐derivatives and their corresponding acetates have an identical elution order compared to pentyl‐homologs ((S)‐HHCP before (R)‐HHCP, compare Figure 2). An identical elution order (first (S)‐ then (R)‐diastereomer) is also assumed for H4CBD, since the second peak consistently had a stronger signal. The assignment of Δ^9^‐ and Δ^8^‐THCP, however, was more complex due to the occurrence of an additional isomer. A further THC‐isomer was also observed in some seizures, so that an assignment analogue to the pentyl‐homologs was assumed (Figure 4). For HHCP and HHCP‐O, mass transitions and elution order were re‐verified after receipt of reference substances. Acetates Δ^8^‐ and Δ^9^‐THC resp. Δ^8^‐ and Δ^9^‐THCP could not be separated from each other using these chromatographic conditions.

### 3.3 Analysis of Seizure Collective

In terms of product types, the seizure collective mainly consisted of cannabis flowers (70.0%, *n* = 56) and resin (16.3%, *n* = 13) whereas vape liquids (2.50%, *n* = 2), edibles (gummies, 6.25%, n = 5) and papers (5.00%, *n* = 4) made up a lower fraction.

#### 3.3.1 Quantitative Results

**FIGURE 5:**
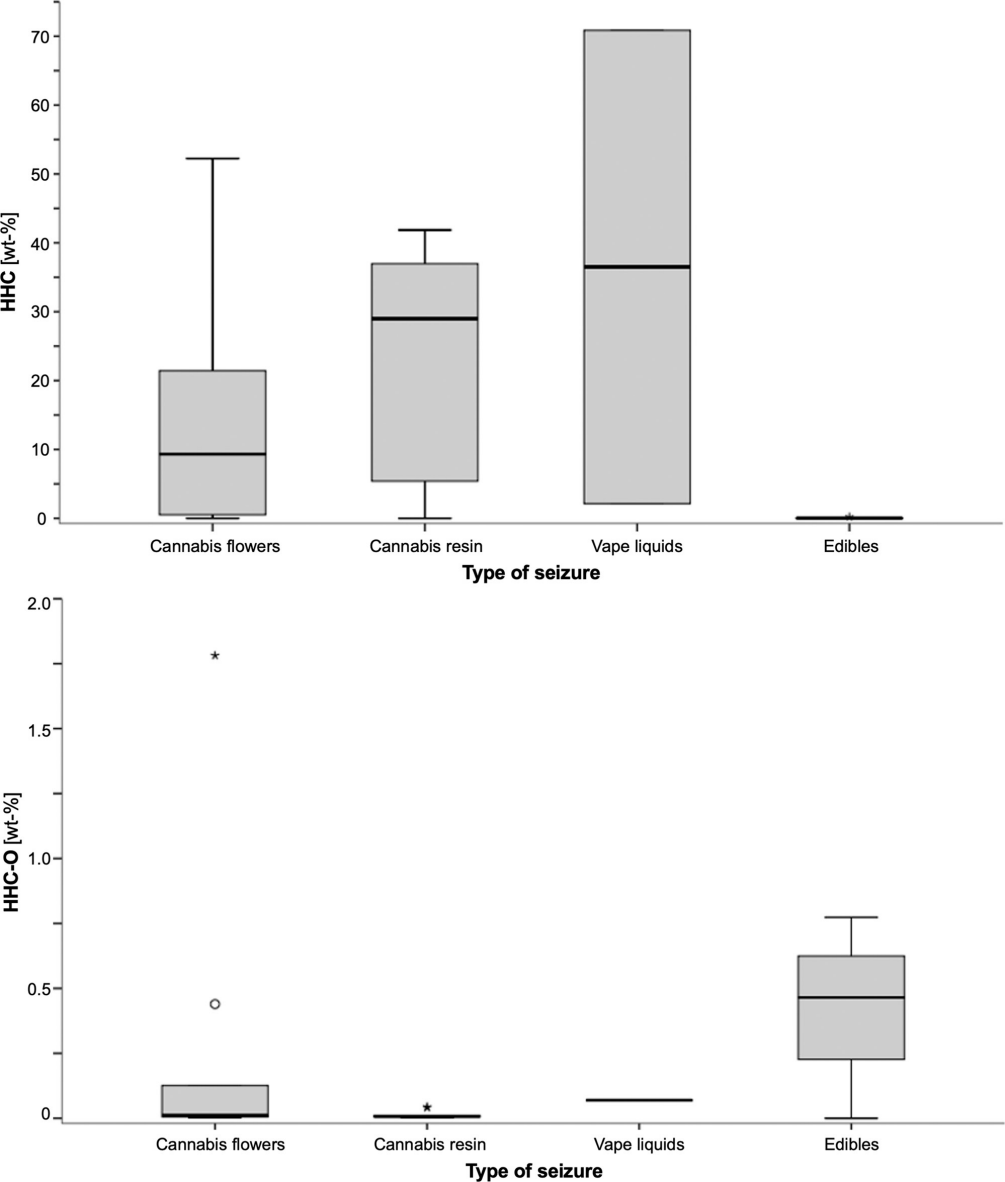
Boxplot of HHC‐ and HHC‐O content (in wt‐%) in the seizure collective. Contents are given as sum of (R)‐ and (S)‐diastereomers.

**FIGURE 6:**
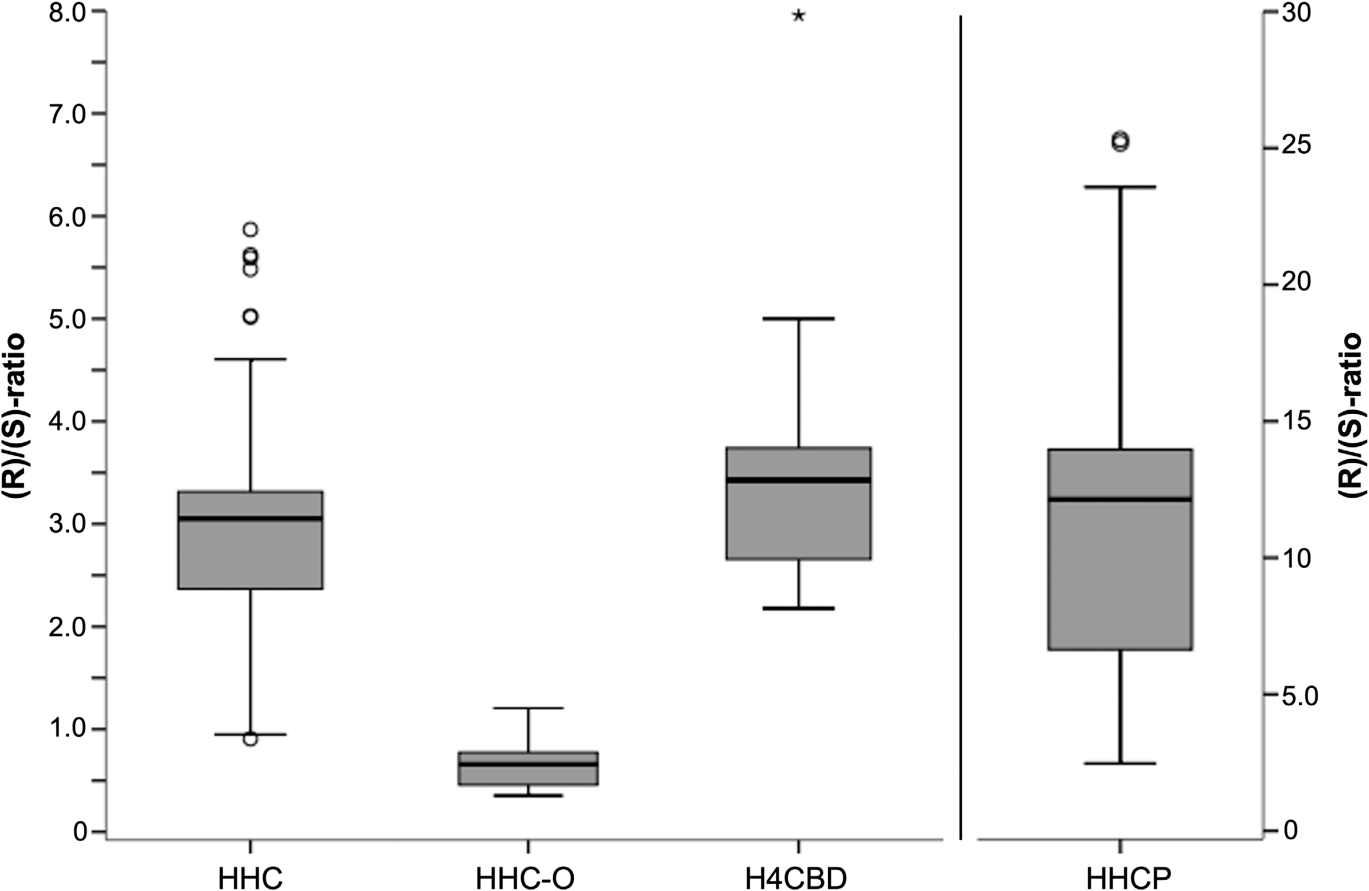
(R)/(S)‐ratios of HHC, HHC‐O, H4CBD and HHCP.

Although with large fluctuations, HHC could be detected in all samples (Figure 5). HHC‐contents of flowers (range < LOQ–52.3 wt‐%, median 9.32 wt‐%) and resin (range 0.00161–41.9 wt‐%, median 29.0 wt‐%) showed no significant differences (Mann–Whitney *U*‐Test *p* > 0.05). The two vape liquids contained either 2.14 wt‐% or 70.9 wt‐% HHC, the latter also being the highest HHC‐content measured in the entire collective. HHC‐content of gummies were between 0.00698–0.202 wt‐%. Papers were only analysed qualitatively, and HHC was detected in all of them. (R)/(S)‐ratios of HHC‐diastereomers were between 0.907–5.87 (median 3.00) in the overall collective (Figure 6). HHC‐O was detected in almost half of the samples (48.8%), including 41.1% of the flowers, 69.2% of the resins, both vape liquids and all edibles. In most of the samples, however, only trace amounts were found (< LOQ in 38.5% of all positive cases), while only six samples (three flowers and three gummies) contained levels > 0.1 wt‐%. (R)/(S) ratios of HHC‐O were significantly lower than those of HHC (range 0.353–1.21, median 0.659, Mann–Whitney *U*‐test *p* < 0.05, Figure 6).

Δ^9^‐THC could be detected in majority of samples (97.5%) including all cannabis flowers (range 0.0281–0.916 wt‐%, median 0.216 wt‐%) and resins (range 0.0119–2.13 wt‐%, median 0.239 wt‐%) with no significant differences in its content (Mann–Whitney U test *p* > 0.05). The Δ^9^‐THC‐contents detected in the two vape liquids were 0.000605 wt‐% and 0.414 wt‐%. Three of five gummies contained traces of Δ^9^‐THC (range < LOQ – 0.000657 wt‐%). Only the neutral form of Δ^9^‐THC was detected in vape liquids, edibles and papers, while the majority of flowers and resins also contained significant amounts of the acidic form Δ^9^‐THCAA (Table S3).

**FIGURE 7:**
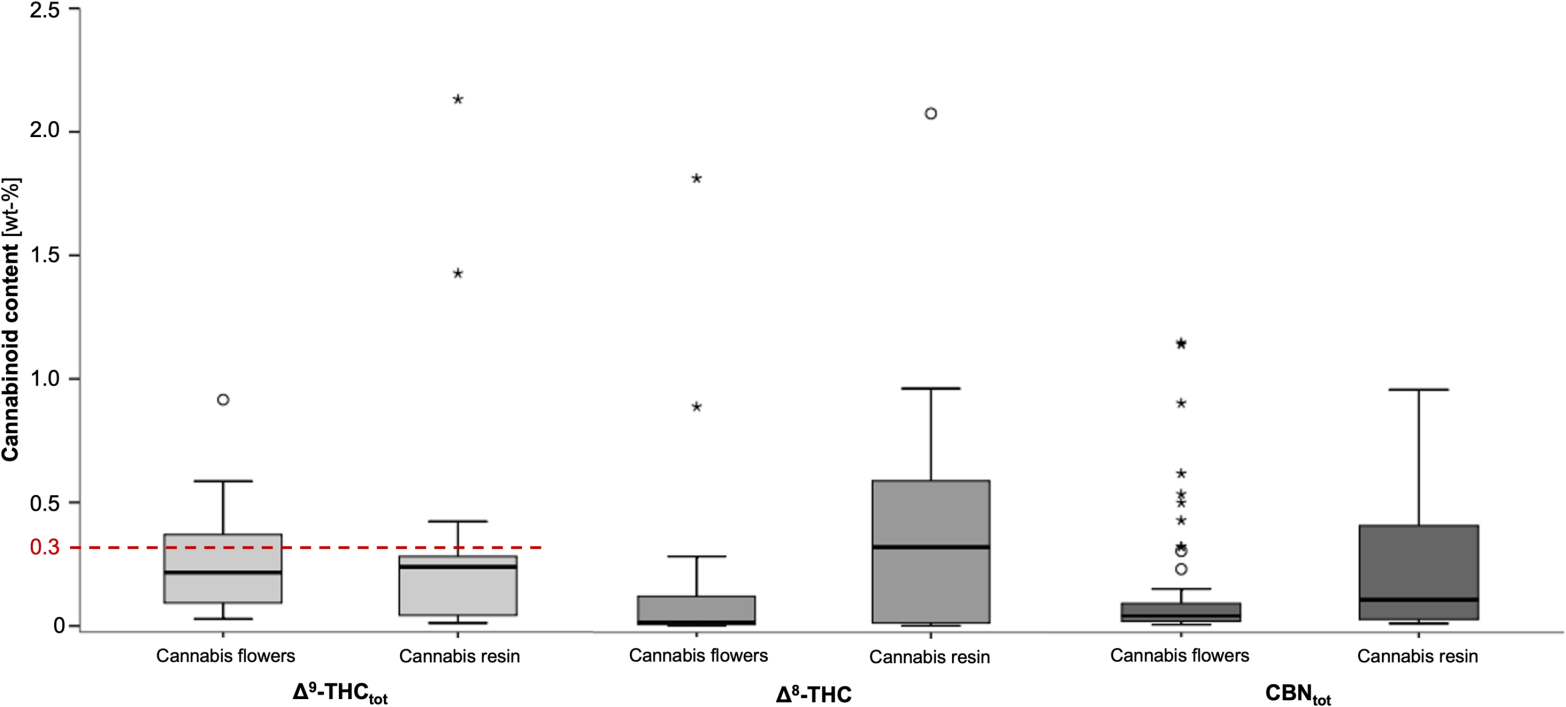
Boxplot of Δ^9^‐THC‐, Δ^8^‐THC‐ and CBN‐contents (in wt‐%) in cannabis flowers and resins. Δ^9^‐THC_tot_ and CBN_tot_ express the total sum value of neutral and acidic form. The Δ^9^‐THC legal limit for hemp according to EU‐regulations (0.3 wt‐%) is highlighted.

Δ^8^‐THC was detected in nearly all of the samples (90.0%) except for six flowers and two gummies. Δ^8^‐THC‐content in resins (range 0.00244–2.08 wt‐%, median 0.320 wt‐%) were significantly higher than in flowers (range 0.00254–1.81 wt‐%, median 0.0169 wt‐%, Mann–Whitney U‐test *p* < 0.05, Figure 7). Vape liquids contained either 0.00312 or 0.433 wt‐% of Δ^8^‐THC. Only minor traces of Δ^8^‐THC were found in gummies (range 0.000199–0.000609 wt‐%). Altogether, the Δ^8^‐THC‐content was > 0.3 wt‐% in 13.2%, > 0.5 wt‐% in 7.89% and > 1.0 wt‐% in 2.63% of the quantified samples.

CBN was found in all flowers (range 0.00721–1.15 wt‐%, median 0.0423 wt‐%) and resins (range 0.0114–0.955 wt‐%, median 0.107 wt‐%) with no significant differences in its content (Mann–Whitney *U*‐test *p* > 0.05, Figure 7). Likewise Δ^9^‐THC, only the neutral form of CBN could be detected in vape liquids (0.000314 resp. 0.164 wt‐%), gummies (range < LOQ – 0.00242 wt‐%) and papers. In 18.4%, 9.21% resp. 2.63% of all quantitatively analysed samples, CBN‐content exceeded 0.3 wt‐%, 0.5 wt‐% resp. 1.0 wt‐%. Traces of CBN‐O were detected in 11.3% of samples (range < LOQ ‐ 0.0840 wt‐%) and exclusively in combination with HHC‐O.

**FIGURE 8:**
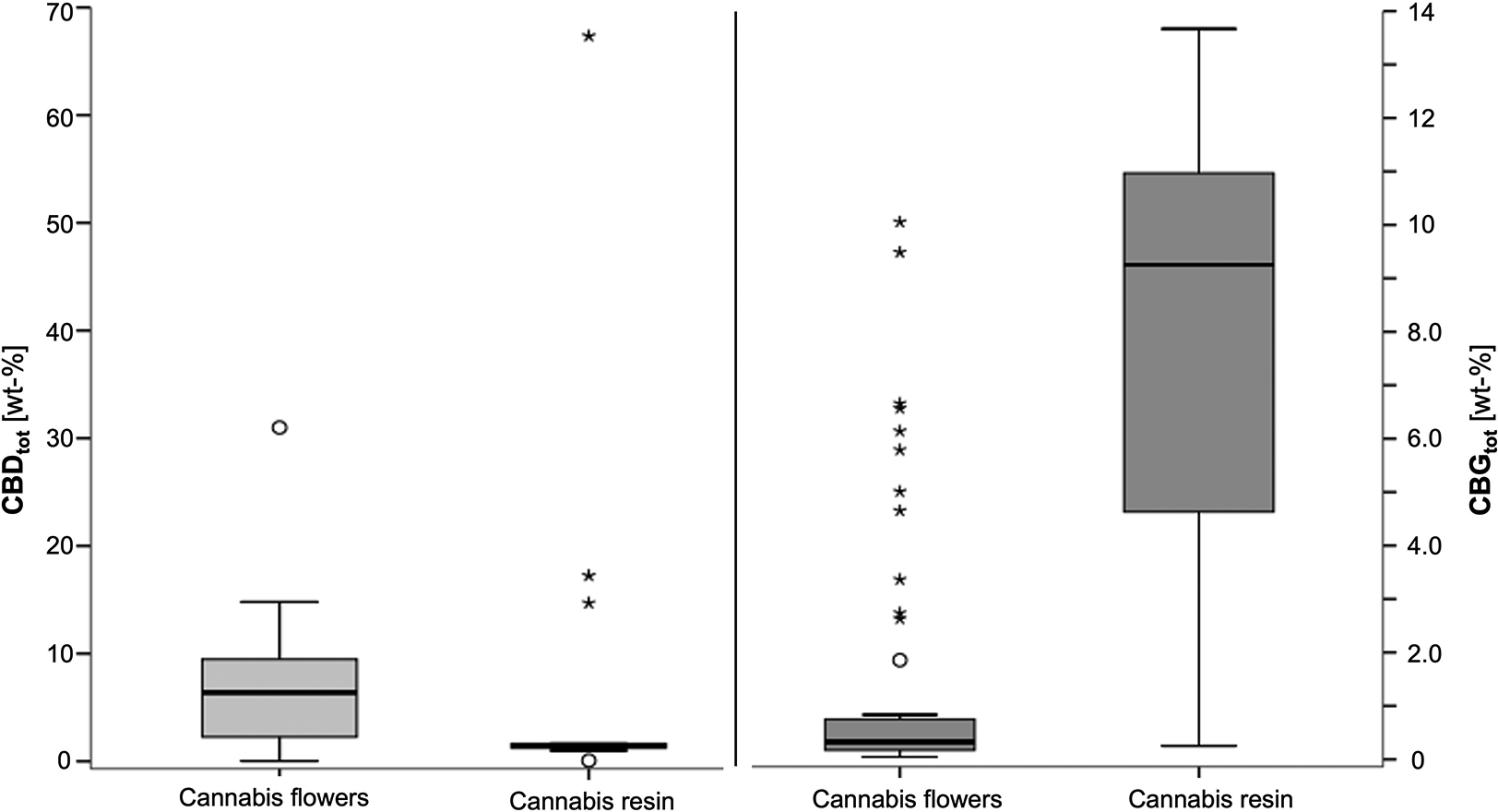
Boxplot of CBD‐ and CBG‐contents (in wt‐%) in cannabis flowers and resins. CBD_tot_ and CBG_tot_ express the total sum value of neutral and acidic form, calculated as neutral form.

Moreover, CBD and CBG could be detected in all flowers (CBD: range 0.0109–31.0 wt‐%, median 6.38 wt‐%, CBG: range 0.0416–10.1 wt‐%, median 0.324 wt‐%) and resins (CBD: range 0.0635–67.3 wt‐%, median 1.42 wt‐%, CBG: range 0.250–13.7 wt‐%, median 9.25 wt‐%, Figure 8). Interestingly, in 19.6% of cannabis flowers and even 69.2% of resins higher CBG‐ than CBD‐contents were observed. Traces of CBD and CBDA were observed in vape liquids (CBD_tot_ 0.00253 resp. 0.0732 wt‐%) and all gummies (range < LOQ – 0.000805 wt‐%). Papers were all positive for CBD. CBGA was only detected in minor amounts in the vape liquid with the highest HHC‐content and in the H4CBD gummies (0.00406 resp. 0.000106 wt‐% calculated in its form as CBG).

#### 3.3.2 Qualitative Results

Apart from HHC, H4CBD was the most frequently detected SSC in about half of the samples (52.5%), including 55.4% of flowers, 69.2% of resins, the separately bought H4CBD‐gummies and one vape liquid. The (R)/(S)‐ratios of H4CBD‐diastereomers were calculated from peak areas (range 2.18–7.96, median 3.43, Figure 6) and showed no significant differences to those of HHC (Mann–Whitney *U*‐test *p* > 0.05). HHCP was detected in 43.8% of samples, among them 46.4% of flowers, 53.8% of resins and both vape liquids. (R)/(S)‐ratios of HHCP‐diastereomers were significantly higher than those of HHC (range 2.47–25.3, median 12.1, Mann–Whitney *U*‐test *p* < 0.05, Figure 6). Except for two flower samples, Δ^9^‐THCP (47.5% of samples, among them 58.9% of flowers and 38.5% of resins) was always detected in combination with Δ^8^‐THCP (45.0% of samples, among them 55.4% of flowers and 38.5% of resins), with Δ^9^‐THCP being the predominant isomer in the majority of samples (80.6%).

HHCP‐O was detected in 36.3% of the samples (39.3% of flowers, 30.8% of resins and three gummies). With the exception of one sample (No. 49, Table S4), HHCP‐O occurred either in combination with HHCP or HHC‐O. The vast majority of the samples showed only weak signals for HHCP‐O in 1:1,000 dilutions. However, seven flowers were so highly concentrated that they had to be measured again in 1:10,000 dilutions due to saturation (approx. 1,000‐fold enhanced compared to other samples). Traces of THCP‐O were detected in exclusively these seven flowers (8.75% of sample collective). Besides THCP‐O, THC‐O was one of the least frequently detected derivatives (13.8%) and only occurred in combination with HHC‐O. No SSC‐derivates at all were detected in papers.

## 4 Discussion

### 4.1 Validation

In the present study, a LC–MS/MS‐based method for quantitative and qualitative analysis of selected semi‐synthetic and natural cannabinoids was validated and applied to a seizure collective. LC–MS/MS analysis was performed on a classical C18‐column using methanol as organic mobile phase while fulfilling all validation parameters. A step gradient elution was necessary to allow the detection of the early eluting cannabinoid acids in combination with a baseline separation of HHC‐diastereomers. The co‐elution of CBL and Δ^8^‐THC was classified as negligible interference in terms of selectivity, since in previous studies, the neutral form of CBL was either detected not at all [35] or usual levels were extremely low (< 0.0009 wt‐%) [34]. Moreover, since CBL is regarded as an oxidative decomposition product of CBC, it is of minor relevance with regard to its natural occurrence in the cannabis plant [36].

By using three separate calibration solutions for cannabinoid acids, neutral and acetylated cannabinoids, it could easily be ruled out that there were any interferences in the quantification of the neutral cannabinoids by decarboxylations or deacetylations. The derivatisation of the reference substances to acetate has already been used for quantitative analyses [28]. Nevertheless, the completeness of the conversion to acetates was checked with reference to the calibration of the neutral cannabinoids. A 100 ng/mL acetate‐solution yielded values < LOQ for (R)‐ resp. (S)‐HHC whereas CBN was not even detectable. Based on standard addition, no matrix effects were observed for sample types flowers, resins and gummies. The two vape liquids were not further tested for matrix effects, as it can be assumed that these are already volatile alcoholic solutions similar to the extraction solvent methanol. Sample No. 75 (ρ = 0.76 g/mL, Table S2), for example, had a comparable density to methanol (ρ = 0.79 g/mL at 25°C [37]).

### 4.2 Qualitative Identification of SSC‐Derivatives

(R)‐ and (S)‐HHCP, Δ^8^‐ and Δ^9^‐THCP, Δ^8^‐ and Δ^9^‐THC‐O, (R)‐ and (S)‐HHCP‐O, Δ^8^‐ and Δ^9^‐THCP‐O as well as (R)‐ and (S)‐H4CBD could be successfully implemented into the methodology for qualitative screening and the observed mass transitions can be explained plausibly. For example, the dominant signals in the spectra of H4CBD [38] and THCP [39] match previously published LC–MS/MS data. The spectrum of H4CBD is characterised by only a few dominant fragments, most of which had mass‐to‐charge ratios below 100. The H4CBD‐fragment with m/z = 139 was previously described as typical corresponding to the saturated terpene moiety (analogous to m/z = 137 in HHC and HHCP, or m/z = 135 in THC, THCP and CBD) [10], while the fragment m/z = 181 is proposed to correspond to olivetol and m/z = 83 to a residue of the terpene moiety [38]. The characteristic fragment m/z = 221 of heptyl‐derivatives has a shift of m/z = +28 (+C_2_H_4_) due to the prolonged sidechain, corresponding to m/z = 193 in pentyl‐homologs. Mass spectra of acetates typically exhibit a deacetylated fragment corresponding to the mass of the neutral cannabinoid as well as a similar fragmentation to the latter. Although Δ^8^‐ and Δ^9^‐THC‐O resp. Δ^8^‐ and Δ^9^‐THCP‐O could not be separated from each other, these analytes were of minor importance and only trace amounts were rarely detected. The elution order of isomers appears to be consistent between pentyl‐ and heptyl‐homologs which increases the reliability of correct identification of SSC‐derivatives even without a reference substance. While the assignment of the HHCP‐diastereomers was in fact confirmed by a reference substance, the identification of Δ^9^‐ and Δ^8^‐THCP was more difficult due to the occurrence of an additional isomer. A corresponding isomer, however, was also observed for pentyl‐analogues eluting prior to Δ^9^‐THC on an octadecyl‐column making it likely that this signal may be attributed to a synthesis by‐product. Corresponding isomeric by‐products, including (6aR,9S)‐Δ^10^‐THC, 9(R,S)‐Δ^6a,10a^‐THC [40] and several iso‐tetrahydrocannabinols [41, 42], have already been described. While the identity of the THC‐ and THCP‐isomer observed here is unknown, at least the constitutional isomers CBC and CBL can be excluded based on the selectivity experiments in the validation procedure. Furthermore, (6aR,9S)‐Δ^10^‐THC and 9(R,S)‐Δ^6a,10a^‐THC seem unlikely, as they elute after Δ^8^‐THC under similar chromatographic conditions on a C18‐column [40, 43].

### 4.3 Analysis of Seizure Collective

#### 4.3.1 Potency: HHC, Δ^9^‐THC, Δ^8^‐THC and CBN

Similar to previous data, HHC‐content was characterised by extreme fluctuations, ranging from < LOQ in three flowers up to 70.9 wt‐% in a vape liquid. Four seized samples, analysed in a further study, contained 9.75–38.1 wt‐% HHC [10]. In a second study, the evaluation of online certificates for over 60 products resulted in HHC‐contents between 15 and 70 wt‐% [44]. These large differences in the strength of the products, which may be further favoured by the uncontrolled application mode of HHC onto the products by spraying, can pose a risk to consumers [26, 44]. Surprisingly, also the H4CBD‐gummies contained HHC. Although only traces (0.0278 wt‐%) were found, this may be of concern as H4CBD, unlike HHC, does not activate the CB1‐receptor [25]. Ingestion of a higher amount of gummies may therefore still lead to unexpected psychoactive effects. Except for one flower (0.907) and one resin sample (0.947), (R)/(S)‐ratios of HHC‐diastereomers were consistently > 1 with a median of 3.00. The frequent excess of the (R)‐diastereomer is in line with previous studies describing ranges from 1.35–2.28 [10], 0.2–2.4 (average 1.4) [44], 1.13–3.76 [25] and 0.9–1.6 [45]. It can be assumed that an excess of (R)‐HHC is favoured, as it is about 10 times more potent and about 3 times more effective than the (S)‐diastereomer [25].

Δ^9^‐THC was detected in the majority of samples, with 30.4% of flowers, 23.1% of resins and one of the vape liquids exceeding the EU‐legal Δ^9^‐THC limit for hemp (> 0.3 wt‐%) [46, 47]. HHC is usually produced from CBD through the intermediates Δ^8^‐ and Δ^9^‐THC. Exceeding the legally permitted Δ^9^‐THC‐level has already been reported for products containing Δ^8^‐THC sold in the USA [7]. The ratio of Δ^8^‐ and Δ^9^‐THC produced from CBD depends on the reaction conditions of the acidic cyclisation, with the reduction of Δ^8^‐THC producing higher amounts of the more potent (R)‐HHC [10, 11, 44]. The question arises which proportions of Δ^9^‐THC originate from the natural content and which could be attributed to synthesis residues [10]. An isolated detection of the neutral form in non‐herbal materials such as edibles, vape liquids and papers is highly likely regarded as synthesis residue. In flowers and resins, the separate analysis of acid and neutral form may be helpful, as it can be assumed that only the neutral form is fortified due to synthesis impurities. Accordingly, in the majority of samples (80.4% of the flowers, 92.3% of the resins) the neutral form clearly dominated (up to 13.9 times higher in flowers, up to 22.2 times higher in resins) compared to the acid content. However, the decarboxylation progress of the acid is strongly dependent on storage conditions [36], which are usually unknown in the case of seizures. Similar observations as for Δ^9^‐THC were also made for its oxidation product CBN, making the previously described considerations transferable. In contrast, traces of Δ^8^‐THC, as detected in most samples, are very likely to be interpreted as synthesis residues. Although there is little quantitative data on the natural content of Δ^8^‐THC in the cannabis plant, Δ^9^‐THC‐dominant chemotypes usually contain < 0.1 wt‐% [48], meaning that a significantly lower natural content in low‐THC varieties can be assumed. Elevated Δ^8^‐THC‐levels were rarely found (> 1.0 wt‐% in 2.63%) which could indicate an incomplete reduction from THC to HHC.

#### 4.3.2 Derivatives: H4CBD, Heptyl‐Analogs and Acetates

Apart from HHC, H4CBD was the most frequently detected SSC‐derivate. On the one hand, H4CBD could be added intentionally. In the past, products have been sold as ‘blends’ and the data collected here underline that seizures can in fact contain diverse mixtures of different SSC. On the other hand, H4CBD could also be formed as a by‐product from remaining CBD in the second HHC‐synthesis step by hydrogenation. The observed similarities between the (R)/(S)‐ratios of HHC and H4CBD may indicate an analogous synthesis process. In line with this assumption, a saturation of CBD with hydrogen is described in literature, which resulted in H4CBD in an (R)/(S)‐ratio of 2.39 [49].

After H4CBD, heptyl‐derivatives were second most frequently detected. Careful monitoring is advisable as the increased lipophilicity is associated with increased potency compared to pentyl‐analogs [25, 26, 50]. Like potency, also complexity of their synthesis is increasing. In contrast to HHC, HHCP is most likely not produced from CBD anymore, but rather fully synthesised by condensing a terpenoid‐moiety with a resorcinol [7, 50] resp. a cyclohexadione [16]. This way, either CBD‐analogs with altered side chain length could be produced and then converted into HHCP [7] or HHCP could be produced directly [16]. Depending on the synthesis method, the (R)/(S)‐ratios of HHCP can differ significantly. Highly variable ratios in the range of 0.5–5.0 have already been reported [45]. Especially the direct reaction offers a higher degree of stereocontrol by choosing a stereospecific terpene precursor, which can explain the comparatively high (R)/(S)‐ratios (median 12.1) observed in this study. Targeting the synthesis on (R)‐HHCP may, analogous to (R)‐HHC, be explained by its up to 10 times higher potency compared to the (S)‐diastereomer [25, 26]. Presumably, fully synthetic production also applies to THCP, since its natural content in Δ^9^‐THC‐dominant chemotypes is very low (range 0.0023–0.0136 wt‐%) [39]. Δ^9^‐THCP was usually detected in combination with Δ^8^‐THCP, which may indicate a synthesis route by acidic cyclisation of a CBD‐analog. The detection of such a CBD‐analog (cannabidiphorol, CBDP) in THCP‐containing products has already been described [38]. So far, only very little data is available on quantitative analysis of THCP in seizures. However, a few samples have already been reported in which the Δ^9^‐THCP content was consistently too low (up to 1,500 times) [38]. A similar observation was made regarding the HHC‐content in one flower (No. 1 containing 0.300 wt‐% HHC and 0.440 wt‐% HHC‐O, labelled with 20% HHC) and one gummie (No. 73, 0.202 wt‐% HHC, labelled with 250 mg HHC), emphasising once again the unpredictable SSC‐levels in products.

Acetylated cannabinoids were of minor relevance in the collective. HHC‐O and HHCP‐O were mostly only detected in trace amounts, so that impurities during production or cross‐contamination before confiscation might be taken into account. Elevated levels of HHC‐O (> 0.1 wt‐%) were only detected in six samples, while HHCP‐O was the main active ingredient in seven flowers. Particularly in relation to three gummies, HHC‐O contributes to a significant proportion of the total HHC‐content. Assuming that a gummy weighs 2 g, the samples contain only 0.140, 0.180 resp. 0.803 mg as free HHC. Taking the acetate into account, however, total HHC‐quantities of 8.14, 13.8 resp. 9.2 mg (calculated as total in its free form) would be ingested. One of these gummies (No. 72) was also mislabelled with ‘THC 250 mg’. CBN‐O and THC‐O only occurred in combination with HHC‐O, which could indicate that an HHC‐extract with corresponding impurities was acetylated. Similarly, THCP‐O was only detected in traces in the seven flowers with elevated HHCP‐O content. A surprisingly low (R)/(S)‐ratio for HHC‐O was found, which was < 1 in the majority of samples. As the acetates have only a minor effect on the CB1‐receptor, they probably act as prodrugs [26]. Accordingly, an excess of (R)‐HHC‐O would also have been expected. However, it should be emphasised that due to predominantly low contents, further data collection is recommended. In contrast to HHC‐diastereomers, the slopes of the calibration curves of both HHC‐O‐diastereomers differed considerably. Therefore, a further evaluation of the (R)/(S)‐ratios of HHCP‐O based on peak area ratios without quantification via reference substances did not appear reasonable.

#### 4.3.3 Carrier Material: CBD‐ and CBG‐Chemotypes

The cannabinoid composition of the plant‐based carrier material of SSC‐containing products has rarely been studied to date. The few data that are available point to predominantly CBD‐dominant plant material [10, 38]. In this study, extreme CBD‐contents were occasionally detected in a flower (No. 7, 31.0 wt‐%) and a resin sample (No. 62, 67.3 wt‐%). Presumably, these samples were artificially enriched with CBD, as a suspicious coating of the flower sample already suggested visually. Likewise, the neutral cannabinoid accounts for the majority of the CBD‐content in both cases. The data collected here also show, for the first time, that the carrier materials can even contain CBG as dominant cannabinoid. CBG, as precursor of the pentyl cannabinoids in the plant [36], is currently advertised as particularly ‘native’ or ‘mother of all cannabinoids’ making a further diversification on the low THC cannabis market beyond CBD towards CBG‐products likely. Although a CBG‐dominant chemotype was described in the literature as early as 1986 [51], it is still surprising that it is already so widespread and readily available (19.6% of flowers; 69.2% of resins). Consistent with the maximum CBG‐content measured here (a resin sample, No. 66: 13.7 wt‐% CBG, 0.0635 wt‐% CBD), cultivars up to approx. 11 wt‐% have been described [36]. The CBG‐dominant chemotype, however, usually contains only small amounts of CBD (about 10%–15% of the total cannabinoid content), which is explained by the fact that the ‘CBG‐allele’ is actually a weakly functional form of CBDA‐synthase, the enzyme which catalyses the conversion of CBGA to CBDA in the plant [52]. This is in conflict with some samples that contain both, CBD and CBG, in relevant proportions (e.g. No. 18: 2.23 wt‐% CBD, 3.36 wt‐% CBG). Unlike the CBD/THC‐balanced chemotype, the breeding of a chemotype with an equal CBD/CBG‐content is more difficult. In contrast to the alleles that dominantly encode CBDA‐ or THCAA‐synthase, the ‘CBG‐allele’ is inherited recessively [52]. Such a cannabinoid constellation can therefore most likely be explained by a ‘blend’ of at least two separate plants, a CBD‐ and a CBG‐dominant one. Less likely, such a cannabinoid profile is produced only by one single plant, as only one cannabis variety (*‘Carma’*) has so far been described in scientific literature, which contains CBG and CBD in a ratio of approx. 6:1 [36, 53]. However, it should be emphasised that, as part of the seizure process, especially in case of larger sample amounts, variety‐specific composite samples were taken in accordance with current guidelines [54]. Thus, it cannot be completely ruled out that additional artificial mixing was caused by the seizure process, but the results indicate that CBD‐ and CBG‐dominant carrier materials are used very interchangeably.

A further limitation of the collective is that several samples stem from one seizure process (79 samples from 20 seizures), some of which are very similar in its composition. While this mainly influences the medians, the ranges still show a good orientation of the products on the market. A ban on HHC and its derivatives through the German New Psychoactive Substances Act (NpSG) was introduced relatively late in June 2024 [55]. The seizures analysed here originate from a time before the ban. The HHC‐ban will probably influence future developments, which may limit the representativeness of the trends observed here.

## 5 Conclusion

The unawareness of constituents in SSC‐containing products due to mislabelling in combination with strongly fluctuating HHC contents or so‐called ‘blends’ containing SSC‐mixtures can pose a risk to consumers. Another characteristic of this new cannabis trend is the use of cannabis carrier material with unusual cannabinoid compositions, including artificially enhanced CBD‐contents, literal ‘designer strains’ with CBG‐dominance or even ‘blends’ of CBD‐ and CBG‐dominant material. Comprehensive monitoring of available products is therefore strongly recommended. Due to the rapid dynamics, keeping analytical methodologies up‐to‐date is a major challenge. A combination of quantification and qualification as presented here may be a viable option to react quickly to newly emerging derivatives. Despite the bans, there are recent reports about a ‘further generation’ of SSCs that are not yet covered by current legislations due to the introduction of hydroxy‐groups (e.g. 10‐OH‐HHC resp. ‘10‐HC’; 10‐OH‐HHCP resp.’10‐HCP’). Finally, the systematic investigation of seizures also provides a valuable data basis for the investigation of biological material.

## Conflicts of Interest

The authors declare no conflicts of interest.

## Supporting information


**Figure S1** Product ion scans of SSC‐derivatives. Depicted collision energies were chosen to show fragmentation as comprehensive as possible. Mass spectrum of seizure No. 2 showing HHCP (co‐elution of (R) and (S)‐diastereomer) at a collision energy of 38 eV (a). Mass spectrum of seizure No. 62 (a resin sample labelled with THCP) showing THCP (co‐elution of Δ^9^‐THCP and traces of Δ^8^‐THCP) at a collision energy of 22 eV (b). Mass spectrum of the separately bought edibles showing H4CBD (co‐elution of (R) and (S)‐diastereomer) at a collision energy of 22 eV (c). Mass spectrum of acetylated seizure No. 59 showing HHCP‐O (co‐elution of (R) and (S)‐diastereomer) at a collision energy of 30 eV (d). Mass spectrum of acetylated seizure No. 28 showing THC‐P (co‐elution of Δ^9^‐THCP and traces of Δ^8^‐THCP) at a collision energy of 30 eV (e). Mass spectrum of Δ^9^‐THC‐O (acetylation of a 10 μg/mL solution Δ^9^‐THC) at a collision energy of 30 eV (f). Mass spectrum of Δ^8^‐THC‐O (acetylation of a 10 μg/mL solution Δ^8^‐THC) at a collision energy of 30 eV (g).
**Table S1.** Descriptions of the seizures. The separately bought H4CBD gummies are marked with (*).
**Table S2.** Standard addition results.
**Table S3.** Raw data ‐ Quantification of HHC, Δ^9^‐THC, Δ^8^‐THC, CBN, CBD and CBG in the seizure collective. Seizures are sub‐divided into groups according to their sample types and numbered. The separately bought H4CBD gummies are marked with (*). Elevated Δ^9^‐THC, Δ^8^‐THC and CBN‐contents are marked (≥ 0.3–≤ 0.5 wt‐%, ≥ 0.5–≤ 1.0 wt‐%, ≥ 1.0 wt‐%). The dominant cannabinoid of the carrier material is highlighted. In some cases, a mixture of CBD‐ and CBG‐dominant carrier material of CBD‐ and CBG‐dominant is to be considered. This was considered when the CBD exceeded 15% of the total cannabinoid content in CBG‐dominant material or when the CBG content was conspicuously high (> 15% of total cannabinoid content) in CBD‐dominant material.
**Table S4.** Raw data ‐ Quantification of HHC‐O and CBN‐O as well as qualitative analysis of further derivatives in the seizure collective. Elevated HHC‐O (> 0.1 wt‐%) and HHCP‐O contents are marked. Isolated detections of HHCP‐O without HHC‐O or HHC‐P are highlighted.

## Data Availability

The data that supports the findings of this study are available in the supplementary material of this article.
