# Supplementary material for: Characterising a New Cannabis Trend: Extensive Analysis of Semi‐Synthetic Cannabinoid‐Containing Seizures From Germany
Source: Drug Test Anal. 2025 Mar 19;17(9):1803–16. doi: 10.1002/dta.3886 (PMC12401644; doi:10.1002/dta.3886)
Supplement: Supplementary file 1 — Figure S1 Product ion scans of SSC‐derivatives. Depicted collision energies were chosen to show fragmentation as comprehensive as possible. Mass spectrum of seizure No. 2 showing HHCP (co‐elution of (R) and (S)‐diastereomer) at a collision energy of 38 eV (a). Mass spectrum of seizure No. 62 (a resin sample labelled with THCP) showing THCP (co‐elution of Δ9‐THCP and traces of Δ8‐THCP) at a collision energy of 22 eV (b). Mass spectrum of the separately bought edibles showing H4CBD (co‐elution of (R) and (S)‐diastereomer) at a collision energy of 22 eV (c). Mass spectrum of acetylated seizure No. 59 showing HHCP‐O (co‐elution of (R) and (S)‐diastereomer) at a collision energy of 30 eV (d). Mass spectrum of acetylated seizure No. 28 showing THC‐P (co‐elution of Δ9‐THCP and traces of Δ8‐THCP) at a collision energy of 30 eV (e). Mass spectrum of Δ9‐THC‐O (acetylation of a 10 μg/mL solution Δ9‐THC) at a collision energy of 30 eV (f). Mass spectrum of Δ8‐THC‐O (acetylation of a 10 μg/mL solution Δ8‐THC) at a collision energy of 30 eV (g). Table S1. Descriptions of the seizures. The separately bought H4CBD gummies are marked with (*). Table S2. Standard addition results. Table S3. Raw data ‐ Quantification of HHC, Δ9‐THC, Δ8‐THC, CBN, CBD and CBG in the seizure collective. Seizures are sub‐divided into groups according to their sample types and numbered. The separately bought H4CBD gummies are marked with (*). Elevated Δ9‐THC, Δ8‐THC and CBN‐contents are marked (≥ 0.3–≤ 0.5 wt‐%, ≥ 0.5–≤ 1.0 wt‐%, ≥ 1.0 wt‐%). The dominant cannabinoid of the carrier material is highlighted. In some cases, a mixture of CBD‐ and CBG‐dominant carrier material of CBD‐ and CBG‐dominant is to be considered. This was considered when the CBD exceeded 15% of the total cannabinoid content in CBG‐dominant material or when the CBG content was conspicuously high (> 15% of total cannabinoid content) in CBD‐dominant material. Table S4. Raw data ‐ Quantification of HHC‐O and CBN‐O as well as qualitative an [file DTA-17-1803-s001.docx]

**Supporting information**

**Characterising a new cannabis trend: Extensive analysis of semi-synthetic cannabinoid-containing seizures from Germany**

Marica Hundertmark^1^, Laura Besch^2^, Jörg Röhrich^1^, Tanja Germerott^1^, Cora Wunder^1^

^1^Department of Forensic Toxicology, Institute of Legal Medicine, University Medical Center

^2^Forensic Science Institute, Dez.33 Chemistry/Toxicology, State Office of Criminal Investigation Rhineland-Palatinate, Mainz, Germany


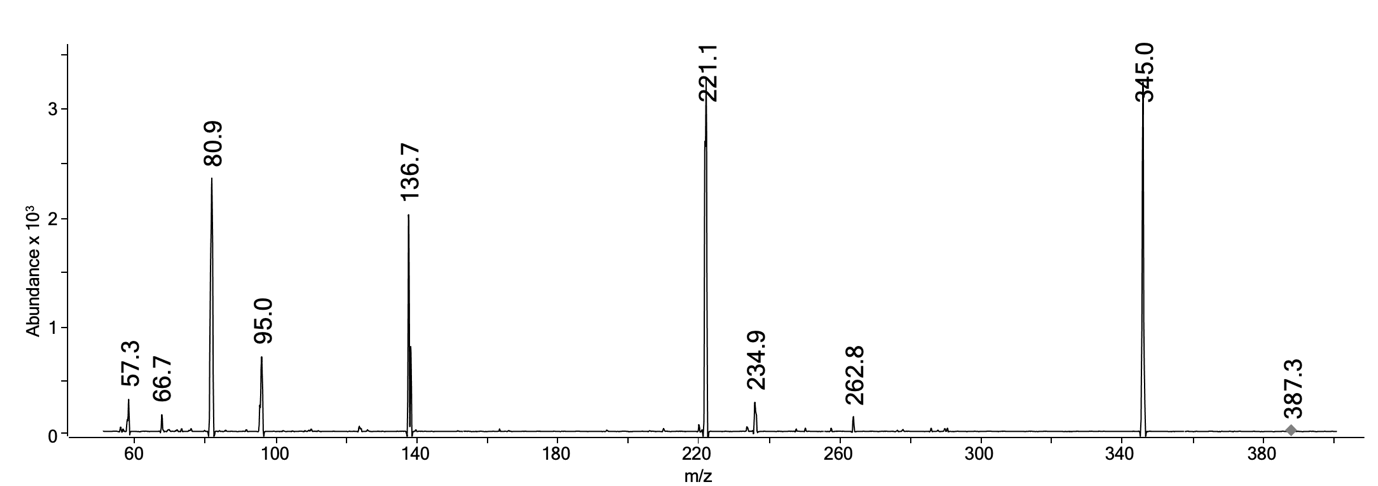


**(d)**


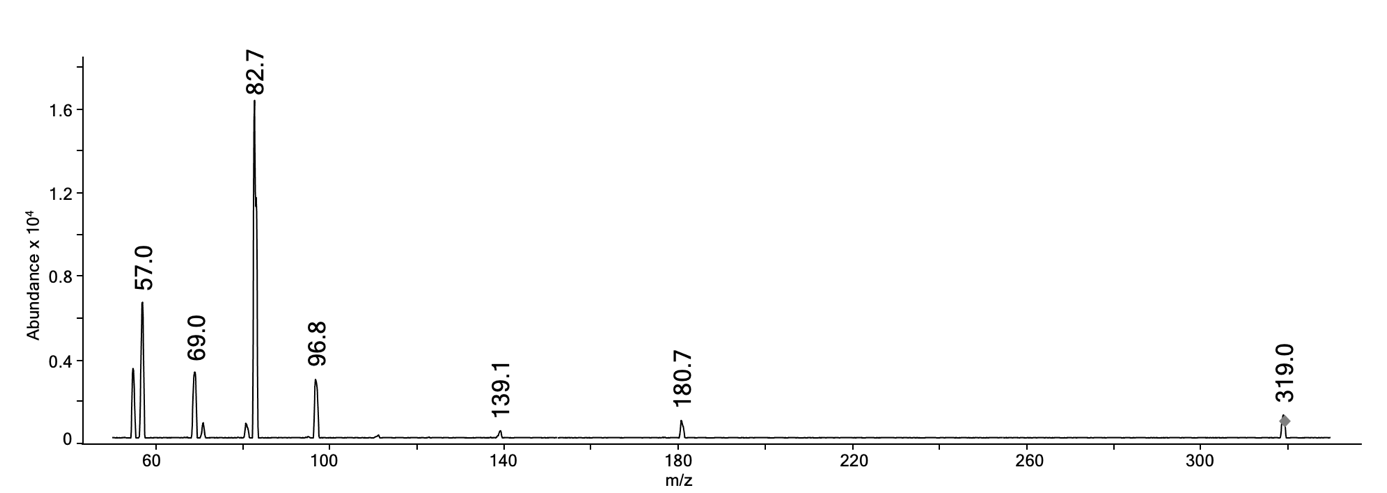

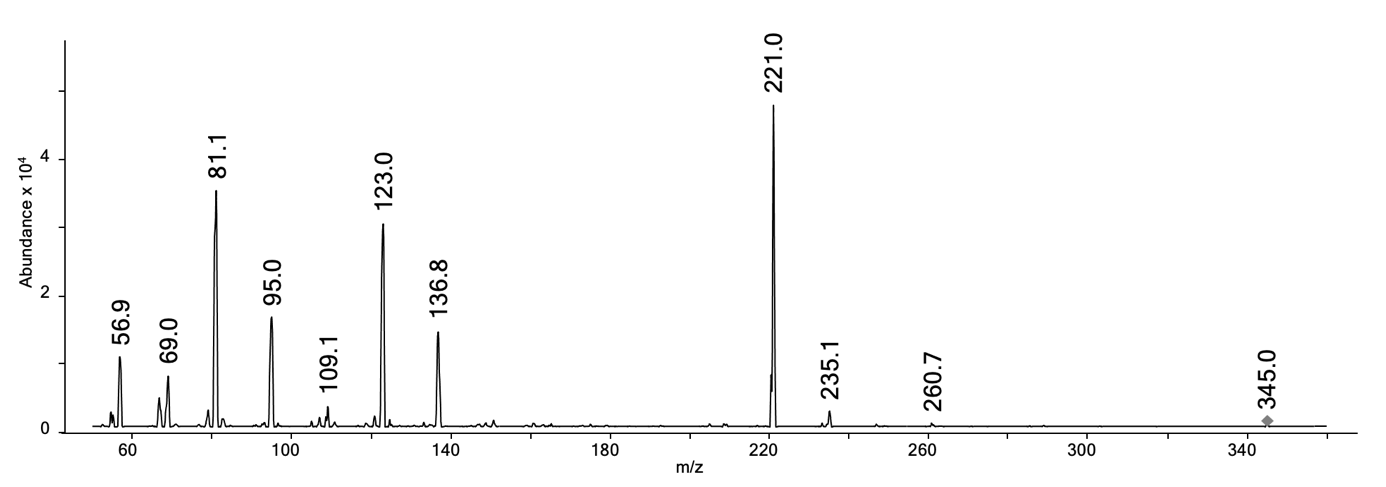

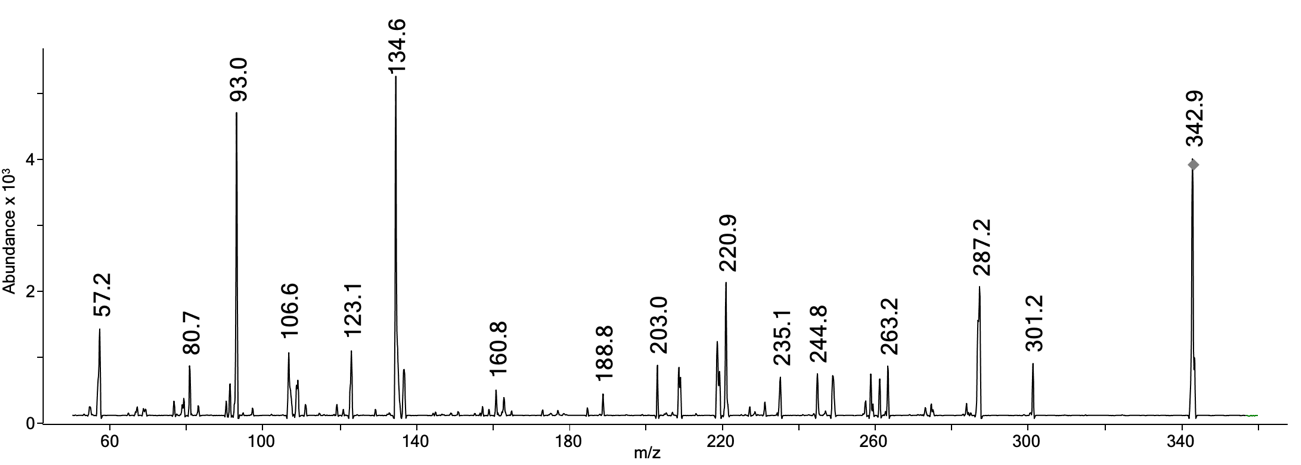


**(a)**

**(b)**

**(c)**


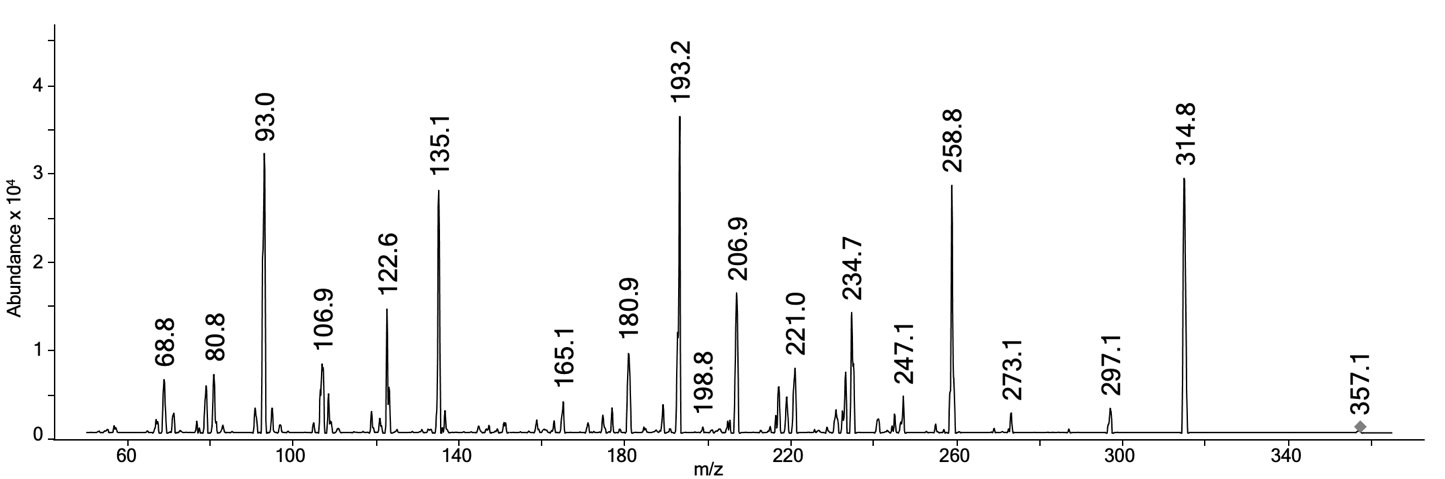

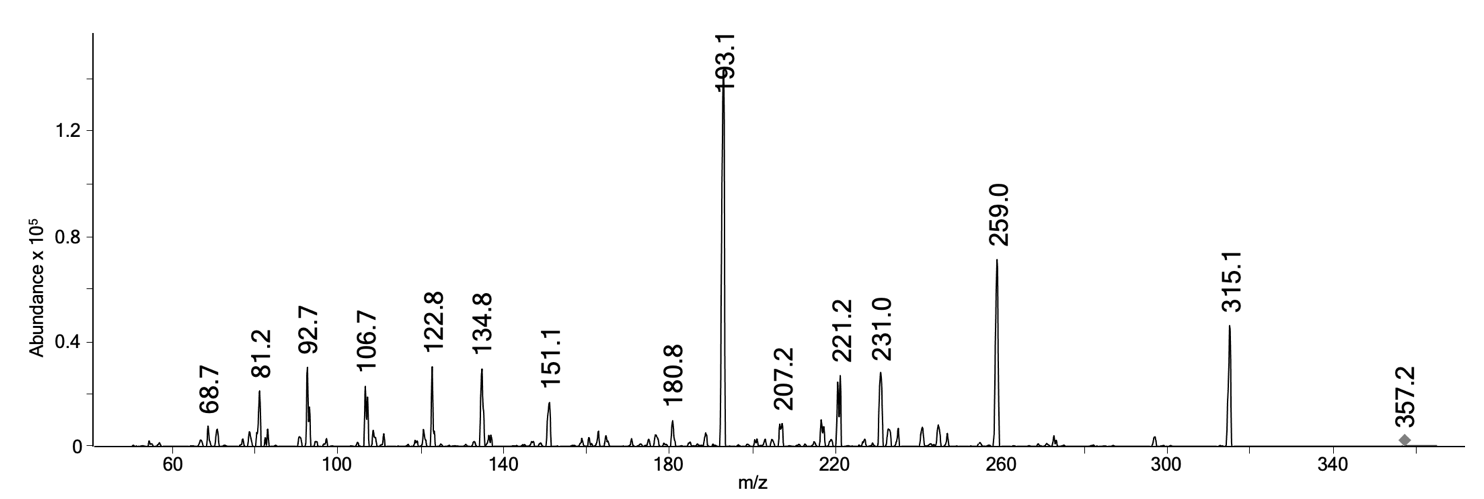

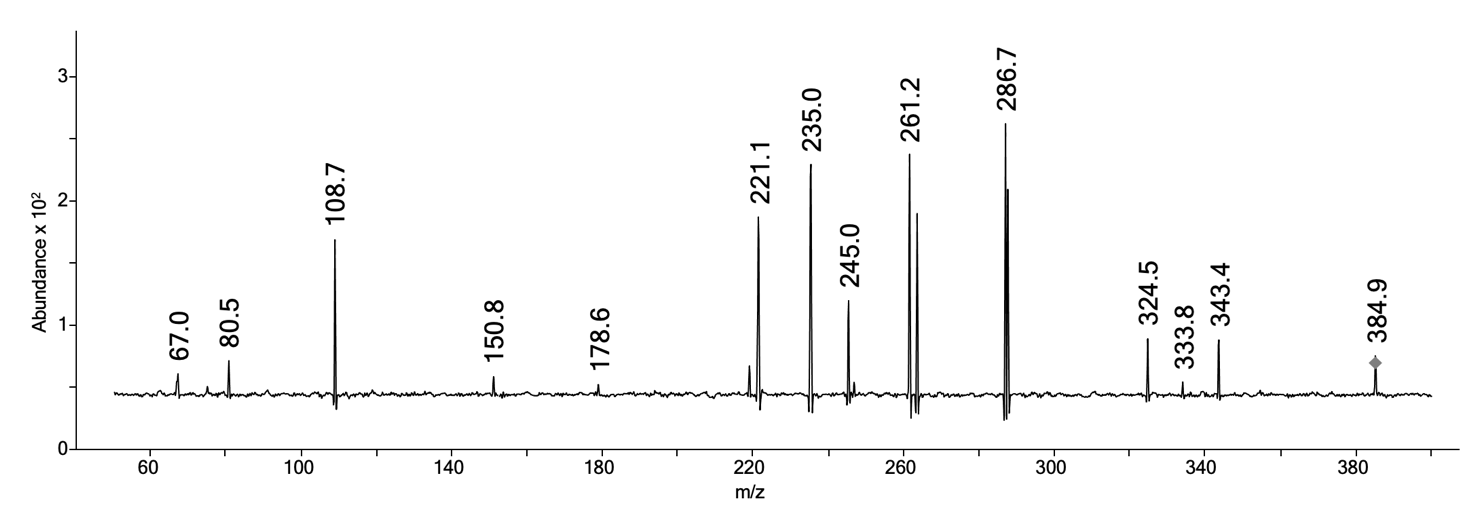


**(g)**

**(f)**

**(e)**

**Figure S1 Product ion scans of SSC-derivatives.** Depicted collision energies were chosen to show fragmentation as comprehensive as possible. Mass spectrum of seizure No. 2 showing HHCP (co-elution of (R) and (S)-diastereomer) at a collision energy of 38 eV (a). Mass spectrum of seizure No. 62 (a resin sample labelled with THCP) showing THCP (co-elution of Δ^9^-THCP and traces of Δ^8^-THCP) at a collision energy of 22 eV (b). Mass spectrum of the separately bought edibles showing H4CBD (co-elution of (R) and (S)-diastereomer) at a collision energy of 22 eV (c). Mass spectrum of acetylated seizure No. 59 showing HHCP-O (co-elution of (R) and (S)-diastereomer) at a collision energy of 30 eV (d). Mass spectrum of acetylated seizure No. 28 showing THC-P (co-elution of Δ^9^-THCP and traces of Δ^8^-THCP) at a collision energy of 30 eV (e). Mass spectrum of Δ^9^-THC-O (acetylation of a 10 µg/mL solution Δ^9^-THC) at a collision energy of 30 eV (f). Mass spectrum of Δ^8^-THC-O (acetylation of a 10 µg/mL solution Δ^8^-THC) at a collision energy of 30 eV (g).

| **Sample No.** | **Seizure No.** | **Total weight [g]** | **Description** |  |
| --- | --- | --- | --- | --- |
| **Cannabis flowers** | | | | |
| 1 | 1 | 4.56 | Two sealed clip bags “HHC COSMIC Skywalker OG...vitalised CBD flowers 20% HHC <0.2% THC...2g” each contain cannabis flowers |  |
| 2 | 2 | 51.8 | "Tangie 51" |  |
| 3 | 2 | 130 | “Lemon King” |  |
| 4 | 2 | 33.4 | Brown coated cannabis flowers, **composite sample** |  |
| 5 | 3 | 1.01 | Sealed black clip bag containing cannabis flowers |  |
| 6 | 4 | 1.10 | “White Cherry”, packed cannabis flowers |  |
| 7 | 5 | 382 | 3 x brown coated cannabis flowers, **composite sample** |  |
| 8 | 6 | 2.09 | Ground, brownish plant substance |  |
| 9 | 7 | 93.1 | “Amnesia” |  |
| 10 | 8 | 62.5 | Ground plant material |  |
| 11 | 7 | 149 | “Northern Lights” |  |
| 12 | 7 | 102 | “Purple Punch” |  |
| 13 | 7 | 197 | “Hammer Haze” |  |
| 14 | 8 | 0.31 | - |  |
| 15 | 8 | 2.05 | - |  |
| 16 | 7 | 97.4 | “Purple Punch” |  |
| 17 | 7 | 97.6 | “Green Crack” |  |
| 18 | 8 | 59.5 | “Amnesia Haze”, **composite sample** |  |
| 19 | 8 | 149 | “New York Diesel”, **composite sample** |  |
| 20 | 8 | 311 | “O.G. Kush”, **composite sample** |  |
| 21 | 8 | 88.8 | “Gorilla Glue”, **composite sample** |  |
| 22 | 8 | 38.0 | “Zkittelz”, **composite sample** |  |
| 23 | 8 | 609 | “Gelatto”, **composite sample** |  |
| 24 | 8 | 1.33 | Ground, brownish, sticky substance |  |
| 25 | 8 | 71.8 | Ground plant material |  |
| 26 | 9 | 4.82 | “Strawberry HHC” |  |
| 27 | 9 | 4.98 | “Lemon Queen” |  |
| 28 | 9 | 4.95 | “Haze Island” |  |
| 29 | 9 | 4.99 | “Lemon King” |  |
| 30 | 8 | 98.0 | “Amnesia” |  |
| 31 | 7 | 68.4 | “Pineapple express” |  |
| 32 | 7 | 98.8 | “Sour Diesel” |  |
| 33 | 10 | 31.5 | - |  |
| 34 | 10 | 48.6 | - |  |
| 35 | 10 | 4.86 | - |  |
| 36 | 10 | 2.41 | “Crazy seven” |  |
| 37 | 10 | 5.32 | - |  |
| 38 | 10 | 0.930 | - |  |
| 39 | 10 | 3.13 | - |  |
| 40 | 10 | 1.35 | - |  |
| 41 | 10 | 0.930 | - |  |
| 42 | 10 | 2.87 | Ground plant material |  |
| 43 | 10 | 0.960 | - |  |
| 44 | 10 | 0.940 | - |  |
| 45 | 10 | 1.62 | “Lemon King” |  |
| 46 | 10 | 1.60 | “Zushi Tsunami” |  |
| 47 | 10 | 2.14 | - |  |
| 48 | 11 | 17.2 | “Cali Jockerz CBD”, 9 packs of 2 g |  |
| 49 | 11 | 20.4 | “Crazy 7 CBD”, 10 packs of 2 g |  |
| 50 | 11 | 11.9 | “Strawberry CBD”, 6 packs of 2 g |  |
| 51 | 11 | 12.2 | “Lemon Queen CBD”, 7 packs of 2 g |  |
| 52 | 12 | 12.4 | “Gelato” |  |
| 53 | 12 | 11.4 | “AK 47” |  |
| 54 | 12 | 11.9 | “Strawberry” |  |
| 55 | 12 | 11.5 | “Watermelon” |  |
| 56 | 12 | 11.9 | “Lemon Diesel” |  |
| **Cannabis resin** | | | |  |
| 57 | 13 | not known | - |  |
| 58 | 2 | 34.3 | Brown resin, **composite sample** |  |
| 59 | 2 | 19.6 | Brown resin, **composite sample** |  |
| 60 | 2 | 33.9 | Brown resin, **composite sample** |  |
| 61 | 14 | 50.2 | Brown resin |  |
| 62 | 15 | 53.1 | Sealed black clip bag “tv bubble hash … THCP 45 %, THC < 0.2 %” containing brown resin |  |
| 63 | 8 | 19.3 | 10 brown-black resin balls, “CBD hash” |  |
| 64 | 8 | 68.8 | “Maroke Hash”, brown resin, **composite sample** |  |
| 65 | 8 | 7.75 | “Temple Balls Hash”, dark-brown resin |  |
| 66 | 10 | 87.8 | Brown-black resin |  |
| 67 | 10 | 96.6 | Brown-black resin |  |
| 68 | 10 | 6.27 | “Hot Blondie”, brown resin |  |
| 69 | 11 | 14.5 | “Nougat CBD”, 8 packs of 2 g, dark-brown resin |  |
| **Edibles** (gummies) | | | |  |
| 70 | 12 | 40.0 | “Strawberry belts”, orange stripe-shaped gummies with packaging |  |
| 71 | 12 | 26.0 | “Million Rope Bites”, brown stripe-shaped gummies with packaging |  |
| 72 | 16 | 38.8 | “Rainbow belts, 500 mg THC”, rainbow-coloured and striped gummies |  |
| 73 | 17 | 391 | “Spacejellys, 250 mg HHC”, 17 packs of multicoloured, fruit-shaped gummy bears, 5 packs were sampled and contents completely homogenised |  |
| * | - | - | “H4CBD-gummies, 42 mg H4CBD”, pack of 3 gummies |  |
| **Vape liquids** | | | |  |
| 74 | 18 | 1.28 | Vape-Liquids ‘HHC’, fruity odour, brownish liquid, **composite sample** |  |
| 75 | 19 | not known | Brownish, clear liquid, slightly viscous, sticky and approx. 0.2 mL filling level in a syringe with scale |  |
| **Papers** (presumably soaked with vape liquid) | | | |  |
| 76 | 20 | not known | 1x DIN A4 sheet (lined, with lettering, glossy surface) |  |
| 77 | 20 | not known | 1x DIN A4 sheet (lined, with lettering, glossy surface) |  |
| 78 | 20 | not known | 1x DIN A4 sheet (lined, with lettering, glossy surface) |  |
| 79 | 20 | not known | 1x DIN A4 sheet (lined, with lettering, glossy surface) |  |

**Table S1 Descriptions of the seizures.** The separately bought H4CBD gummies are marked with (*).

| Analyte | Quantification [ng/mL]  validated method | Standard addition results | | | |
| --- | --- | --- | --- | --- | --- |
|  |  | Quantification [ng/mL] absolute  without ISTD, r^2^ | Deviation [%] | Quantification [ng/mL] relative  with ISTD, r^2^ | Deviation [%] |
| **Seizure No. 2 – cannabis flower** | | | | | |
| (R)-HHC  (1:10,000 dilution) | 53.4 | 40.7  (0.999) | -23.7 | 48.3  (0.996) | -9.43 |
| (S)-HHC  (1:10,000 dilution) | 18.1 | 15.3  (0.999) | -15.4 | 19.3  (0.994) | 6.93 |
| Δ^8^-THC  (1:1,000 dilution) | 58.0 | 45.0  (0.993) | -22.4 | 51.6  (0.998) | 11.0 |
| Δ^9^-THC  (1:1,000 dilution) | 50.8 | 39.8  (0.991) | -21.6 | 45.0  (0.997) | -11.3 |
| CBD  (1:10,000 dilution) | 48.8 | 42.1  (0.999) | -13.9 | 48.9  (0.996) | 0.06 |
| CBG  (1:1,000 dilution) | 34.4 | 28.6  (0.995) | -17.0 | 31.2  (0.996) | -9.24 |
| CBN  (1:1,000 dilution) | 15.3 | 10.8  (0.992) | -29.3 | 16.7  (0.997) | 8.7 |
| Δ^9^-THCAA  (1:10,000 dilution) | 7.99 | 8.81  (0.999) | 10.4 | 8.69  (0.998) | 8.68 |
| CBDA  (1:10,000 dilution) | 348 | 262  (0.992) | -24.8 | 294  (0.992) | -15.6 |
| CBGA  (1:1,000 dilution) | 221 | 173  (0.993) | -21.9 | 231  (0.993) | 4.64 |
| CBNA  (1:1,000 dilution) | 6.71 | 4.71  (0.991) | -29.8 | 5.93  (0.998) | -11.6 |
| (R)-HHC-O  (1:10,000 dilution) | 31.2 | 40.3  (0.998) | 29.5  (0.998) | 39.1  (0.993) | 25.6 |
| (S)-HHC-O  (1:10,000 dilution) | 39.6 | 45.1  (0.991) | 14.2 | 35.8  (0.992) | -9.45 |
| CBN-O  (1:10,000 dilution) | 3.38 | 3.44  (0.996) | 1.69 | 3.71  (1.00) | 9.62 |
| **Seizure No. 61 – cannabis resin** | | | | | |
| (R)-HHC  (1:100,000 dilution) | 73.3 | 57.7  (0.9979) | -21.3 | 65.4  (0.999) | -10.8 |
| (S)-HHC  (1:100,000 dilution) | 18.1 | 14.3  (0.999) | -20.9 | 17.3  (0.999) | -4.69 |
| Δ^8^-THC  (1:10,000 dilution) | 18.3 | 14.3  (1.00) | -21.8 | 15.6  (0.999) | -15.2 |
| Δ^9^-THC  (1:10,000 dilution) | 67.2 | 52.8  (0.998) | -21.5 | 56.5  (0.996) | -15.9 |
| CBD  (1:10,000 dilution) | 50.6 | 46.5  (0.999) | -7.99 | 49.2  (0.998) | -2.70 |
| CBG  (1:10,000 dilution) | 396 | 299  (0.997) | -24.5 | 324  (0.992) | -18.3 |
| CBN  (1:10,000 dilution) | 20.5 | 14.9  (0.995) | -27.4 | 17.1  (0.992) | -16.3 |
| Δ^9^-THCAA  (1:100,000 dilution) | 6.54 | 6.54  (0.997) | 4.60 | 6.67  (0.998) | 2.00 |
| CBDA  (1:10,000 dilution) | 42.9 | 44.8  (0.999) | 4.48 | 48.4  (0.994) | 12.9 |
| CBGA  (1:10,000 dilution) | 206 | 222  (0.995) | 7.75 | 257  (0.993) | 24.4 |
| CBNA  (1:10,000 dilution) | 7.99 | 7.16  (0.998) | -10.4 | 7.82  (0.991) | -2.16 |
| (R)-HHC-O  (1:1,000 dilution) | 1.19 | 1.55  (0.997) | 29.9 | 1.50  (0.999) | 26.5 |
| (S)-HHC-O  (1:10,000 dilution) | 1.85 | 2.19  (0.994) | 18.2 | 2.15  (0.998) | 16.2 |
| **Seizure No. 71 – edibles (gummie)** | | | | | |
| (R)-HHC  (1:1,000 dilution) | 14.5 | 13.2  (0.999) | -9.08 | 14.3  (0.998) | -1.56 |
| (S)-HHC  (1:1,000 dilution) | 6.65 | 5.35  (0.999) | -19.5 | 6.37  (0.999) | -4.21 |
| CBN  (1:1,000 dilution) | 5.37 | 4.76  (0.996) | -11.5 | 4.20  (0.994) | -21.7 |
| (R)-HHC-O  (1:100,000 dilution) | 5.40 | 6.75  (0.998) | 24.9 | 6.71  (0.997) | 24.4 |
| (S)-HHC-O  (1:100,000 dilution) | 11.8 | 9.64  (0.997) | -18.4 | 9.63  (0.995) | -18.5 |
| CBN-O  (1:100,000 dilution) | 1.57 | 1.50  (0.999) | -4.55 | 1.49  (0.999) | -5.36 |

**Table S2 Standard addition results.**

| **#** | (R)-HHC | (S)-HHC | **HHC_tot_** | **(R)/(S)-HHC** | Δ^9^-THC | Δ^9^-THCAA | **Δ^9^-THC_tot_** | **Δ^8^-THC** | CBN | CBNA | **CBN_tot_** | CBD | CBDA | **CBD_tot_** | CBG | CBGA | **CBG_tot_** |
| --- | --- | --- | --- | --- | --- | --- | --- | --- | --- | --- | --- | --- | --- | --- | --- | --- | --- |
| **Cannabis flowers** | | | | | | | | | | | | | | | | | |
| **1** | 0.246 | 0.0535 | **0.300** | **4.61** | 0.196 | 0.0879 | **0.273** | **0.00591** | 0.0402 | 0.00725 | **0.0466** | 2.51 | 5.71 | **7.52** | 0.0668 | 0.158 | **0.205** |
| **2** | 1.30 | 0.449 | **1.75** | **2.90** | 0.119 | 0.199 | **0.293** | **0.144** | 0.0381 | 0.0167 | **0.0527** | 1.21 | 8.66 | **8.81** | 0.0854 | 0.549 | **0.567** |
| **3** | 20.6 | 6.51 | **27.2** | **3.17** | 0.0325 | 0.0392 | **0.0667** | **0.0139** | 0.0389 | 0.00704 | **0.0451** | 0.0900 | 0.377 | **0.421** | 0.190 | 5.09 | **4.65** |
| **4** | 4.79 | 1.96 | **9.24** | **2.18** | 0.0498 | 0.0137 | **0.0618** | **0.0144** | 0.0246 | 0.00119 | **0.0256** | 1.00 | 1.17 | **2.03** | 1.49 | 4.01 | **5.01** |
| **5** | 19.5 | 12.4 | **31.9** | **1.57** | 0.486 | 0.0308 | **0.0756** | **0.00443** | 0.0838 | 0.00159 | **0.0852** | 0.739 | 1.44 | **2.00** | 0.0196 | 0.0466 | **0.0605** |
| **6** | <LOQ | <LOQ | <LOQ | na | 0.0535 | 0.0152 | **0.0669** | nd | 0.00572 | 0.00212 | **0.00758** | 0.976 | 1.49 | **2.29** | 0.0689 | 0.179 | **0.226** |
| **7** | 18.3 | 7.14 | **25.4** | **2.56** | 0.115 | 0.0209 | **0.134** | **0.145** | 0.0603 | 0.00269 | **0.0627** | 30.1 | 1.03 | **31.0** | 0.0314 | 0.0204 | **0.0493** |
| **8** | 19.8 | 21.8 | **41.5** | **0.907** | 0.0643 | 0.0952 | **0.148** | **0.170** | 1.12 | 0.0303 | **1.15** | 1.72 | 3.53 | **4.82** | 0.0825 | 0.857 | **0.835** |
| **9** | 7.88 | 1.44 | **9.32** | **5.48** | 0.374 | 0.0751 | **0.440** | **0.250** | 0.0700 | 0.00911 | **0.0780** | 4.76 | 6.32 | **10.3** | 0.104 | 0.195 | **0.275** |
| **10** | 20.9 | 6.52 | **27.4** | **3.20** | 0.180 | 0.0173 | **0.196** | **0.155** | 0.0145 | nd | **0.0145** | 0.674 | 1.17 | **1.70** | 0.0159 | 0.0617 | **0.0700** |
| **11** | 9.25 | 3.70 | **13.0** | **2.50** | 0.320 | 0.0733 | **0.384** | **0.0684** | 0.314 | 0.00969 | **0.322** | 5.25 | 6.39 | **10.9** | 0.133 | 0.169 | **0.281** |
| **12** | 7.76 | 2.54 | **10.3** | **3.05** | 0.217 | 0.0524 | **0.263** | **0.0413** | 0.131 | 0.00705 | **0.137** | 4.20 | 4.38 | **8.04** | 0.0807 | 0.111 | **0.179** |
| **13** | 15.8 | 6.77 | **22.6** | **2.34** | 0.383 | 0.0761 | **0.450** | **0.207** | 0.297 | 0.00785 | **0.304** | 4.02 | 5.78 | **9.09** | 0.116 | 0.153 | **0.250** |
| **14** | <LOQ | <LOQ | <LOQ | na | 0.0249 | 0.00843 | **0.0322** | nd | 0.00721 | <LOQ | **0.00721** | 0.702 | 0.989 | **1.57** | 0.0269 | 0.0518 | **0.0721** |
| **15** | 39.9 | 12.4 | **52.3** | **3.23** | 0.311 | 0.0234 | **0.330** | **0.889** | 0.148 | 0.00354 | **0.151** | 1.02 | 2.06 | **2.82** | 0.0167 | 0.0284 | **0.0416** |
| **16** | 13.3 | 5.83 | **19.2** | **2.28** | 0.512 | 0.0951 | **0.595** | **0.181** | 0.309 | 0.0127 | **0.321** | 7.18 | 8.17 | **14.3** | 0.203 | 0.194 | **0.373** |
| **17** | 4.68 | 2.22 | **6.90** | **2.10** | 0.711 | 0.234 | **0.916** | **1.81** | 0.0550 | 0.0166 | **0.0695** | 6.31 | 7.81 | **13.2** | 0.295 | 0.484 | **0.721** |
| **18** | 2.02 | 0.624 | **2.65** | **3.24** | 0.0809 | 0.0349 | **0.112** | **0.0175** | 0.0112 | <LOQ | **0.0112** | 0.764 | 1.67 | **2.23** | 0.211 | 3.59 | **3.36** |
| **19** | 9.11 | 3.30 | **12.4** | **2.76** | 0.104 | 0.0205 | **0.121** | **0.0531** | 0.0146 | <LOQ | **0.0146** | 0.967 | 1.70 | **2.46** | 0.0225 | 0.0384 | **0.0562** |
| **20** | 8.29 | 2.62 | **10.9** | **3.17** | 0.113 | <LOQ | **0.113** | **0.0642** | 0.0187 | nd | **0.0187** | 1.54 | 1.96 | **3.04** | 0.0170 | 0.0330 | **0.0459** |
| **21** | 22.2 | 6.73 | **29.0** | **3.30** | 0.194 | 0.0231 | **0.214** | **0.178** | 0.0246 | 0.00322 | **0.0274** | 1.31 | 1.96 | **3.04** | 0.0306 | 0.0419 | **0.0674** |
| **22** | 6.54 | 2.11 | **8.65** | **3.10** | 0.0761 | 0.0187 | **0.0924** | **0.0492** | 0.00838 | nd | **0.00838** | 0.402 | 0.670 | **0.989** | 0.166 | 2.93 | **2.74** |
| **23** | 17.2 | 5.45 | **22.7** | **3.16** | 0.152 | 0.0275 | **0.176** | **0.121** | 0.00922 | nd | **0.00922** | 0.0788 | 0.133 | **0.195** | 0.333 | 6.21 | **5.79** |
| **24** | 13.3 | 6.12 | **19.4** | **2.17** | 0.173 | nd | **0.173** | **0.0721** | 0.0888 | nd | **0.0888** | 1.35 | 0.0733 | **1.41** | 9.13 | 0.404 | **9.49** |
| **25** | 1.38 | 0.466 | **1.85** | **2.97** | 0.0572 | 0.0105 | **0.0664** | **0.0168** | 0.0172 | <LOQ | **0.0172** | 1.44 | 1.63 | **2.87** | 0.0285 | 0.0428 | **0.0661** |
| **26** | 8.79 | 3.62 | **12.4** | **2.43** | 0.124 | 0.107 | **0.216** | **0.0324** | 0.0494 | 0.0132 | **0.0609** | 1.34 | 5.53 | **6.19** | 0.0408 | 0.101 | **0.130** |
| **27** | 0.377 | 0.144 | **0.521** | **2.62** | 0.0582 | 0.0329 | **0.0871** | nd | 0.0166 | 0.00293 | **0.0191** | 0.00876 | 0.0568 | **0.0586** | 0.250 | 11.2 | **10.1** |
| **28** | 0.540 | 0.218 | **0.757** | **2.48** | 0.224 | 0.127 | **0.335** | nd | 0.0237 | 0.00499 | **0.0281** | 2.46 | 7.98 | **9.46** | 0.0889 | 0.240 | **0.299** |
| **29** | 15.1 | 6.68 | **21.7** | **2.25** | 0.0722 | 0.0393 | **0.0772** | nd | 0.230 | 0.00173 | **0.231** | 0.00550 | 0.0422 | **0.0426** | 0.195 | 7.36 | **6.66** |
| **30** | 8.19 | 1.40 | **9.59** | **5.87** | 0.380 | 0.0665 | **0.439** | **0.282** | 0.0719 | 0.00934 | **0.0801** | 4.54 | 6.58 | **10.3** | 0.0915 | 0.125 | **0.201** |
| **31** | 8.66 | 2.55 | **11.2** | **3.40** | 0.396 | 0.0795 | **0.466** | **0.126** | 0.0867 | 0.00993 | **0.0954** | 6.24 | 9.77 | **14.8** | 0.208 | 0.274 | **0.449** |
| **32** | 7.79 | 1.38 | **9.17** | **5.62** | 0.373 | 0.0822 | **0.445** | **0.226** | 0.0752 | 0.0121 | **0.0858** | 5.11 | 7.26 | **11.5** | 0.137 | 0.284 | **0.387** |
| **33** | 7.89 | 2.39 | **10.3** | **3.30** | 0.0201 | 0.0392 | **0.0545** | **0.0443** | 0.0331 | 0.00978 | **0.0417** | 0.0878 | 0.0200 | **0.0263** | 0.181 | 7.27 | **6.57** |
| **34** | 0.333 | 0.110 | **0.442** | **3.02** | 0.0563 | 0.0382 | **0.0898** | **0.00738** | 1.13 | 0.00584 | **1.14** | 1.33 | 2.56 | **3.58** | 0.0669 | 0.528 | **0.530** |
| **35** | <LOQ | <LOQ | <LOQ | na | 0.178 | 0.0263 | **0.201** | **0.00812** | 0.0221 | 0.00186 | **0.0237** | 6.41 | 3.07 | **9.11** | 0.197 | 0.269 | **0.434** |
| **36** | <LOQ | <LOQ | <LOQ | na | 0.222 | 0.0504 | **0.266** | **0.00704** | 0.0241 | 0.00444 | **0.0280** | 5.94 | 4.04 | **9.49** | 0.103 | 0.220 | **0.297** |
| **37** | 0.00807 | 0.00390 | **0.0120** | **2.07** | 0.246 | 0.0608 | **0.299** | **0.00580** | 0.0289 | 0.00702 | **0.0351** | 6.21 | 5.19 | **10.8** | 0.111 | 0.206 | **0.292** |
| **38** | 0.138 | 0.281 | **0.420** | **2.04** | 0.0590 | 0.0469 | **0.100** | **0.00338** | 0.527 | 0.00709 | **0.533** | 1.04 | 2.51 | **3.25** | 0.0487 | 0.140 | **0.172** |
| **39** | 0.199 | 0.0594 | **0.259** | **3.35** | 0.229 | 0.0470 | **0.270** | **0.00980** | 0.0313 | 0.00438 | **0.0352** | 6.71 | 4.25 | **10.4** | 0.209 | 0.410 | **0.569** |
| **40** | 3.51 | 1.11 | **4.62** | **3.17** | 0.210 | 0.0513 | **0.255** | **0.00853** | 0.0392 | 0.00439 | **0.0430** | 5.03 | 3.56 | **8.37** | 0.300 | 1.77 | **1.86** |
| **41** | 0.105 | 0.0210 | **0.126** | **5.02** | 0.0666 | 0.0385 | **0.100** | **0.00398** | 0.493 | 0.00733 | **0.499** | 1.67 | 2.96 | **4.27** | 0.0429 | 0.0839 | **0.117** |
| **42** | 0.198 | 0.0707 | **0.268** | **2.80** | 0.229 | 0.0563 | **0.279** | **0.00927** | 0.0305 | 0.00460 | **0.0354** | 6.82 | 4.65 | **10.9** | 0.204 | 0.566 | **0.701** |
| **43** | 0.253 | 0.109 | **0.362** | **2.33** | 0.0583 | 0.0398 | **0.0932** | **0.00428** | 0.610 | 0.00752 | **0.617** | 1.29 | 2.68 | **3.65** | 0.0481 | 0.132 | **0.164** |
| **44** | 0.138 | 0.0247 | **0.163** | **5.59** | 0.0830 | 0.0342 | **0.113** | **0.00612** | 0.896 | 0.00617 | **0.901** | 1.95 | 3.13 | **4.70** | 0.0676 | 0.119 | **0.172** |
| **#** | (R)-HHC | (S)-HHC | **HHC_tot_** | **(R)/(S)-HHC** | Δ^9^-THC | Δ^9^-THCAA | **Δ^9^-THC_tot_** | **Δ^8^-THC** | CBN | CBNA | **CBN_tot_** | CBD | CBDA | **CBD_tot_** | CBG | CBGA | **CBG_tot_** |
| **Cannabis flowers** (continued) | | | | | | | | | | | | | | | | | |
| **45** | 7.37 | 2.23 | **9.61** | **3.30** | 0.0103 | 0.0203 | **0.0281** | **0.0126** | 0.0202 | 0.00992 | **0.0289** | 0.00386 | 0.00803 | **0.0109** | 0.109 | 6.65 | **5.95** |
| **46** | 0.103 | 0.0313 | **0.134** | **3.30** | 0.0291 | 0.0311 | **0.0563** | **0.00284** | 0.415 | 0.0148 | **0.428** | 1.17 | 2.64 | **3.49** | 0.0420 | 0.0665 | **0.100** |
| **47** | 0.00204 | <LOQ | **0.00204** | na | 0.200 | 0.0563 | **0.249** | **0.00574** | 0.0194 | 0.00507 | **0.0238** | 4.99 | 4.51 | **8.94** | 0.0995 | 0.539 | **0.779** |
| **48** | 5.48 | 2.30 | **7.79** | **2.38** | 0.157 | 0.0778 | **0.226** | **0.0188** | 0.0588 | 0.0112 | **0.0687** | 2.49 | 4.65 | **6.57** | 0.309 | 0.519 | **0.764** |
| **49** | 0.00232 | <LOQ | **0.00232** | na | 0.286 | 0.0805 | **0.356** | nd | 0.0329 | 0.00726 | **0.0392** | 4.41 | 6.37 | **10.0** | 0.184 | 0.397 | **0.533** |
| **50** | 5.88 | 2.43 | **8.31** | **2.42** | 0.268 | 0.137 | **0.388** | **0.0254** | 0.0716 | 0.0154 | **0.0851** | 3.83 | 7.45 | **10.3** | 0.238 | 0.475 | **0.655** |
| **51** | 0.589 | 0.250 | **0.839** | **2.35** | 0.0461 | 0.0540 | **0.0935** | **0.00255** | 0.0273 | 0.00794 | **0.0343** | 0.259 | 1.04 | **1.17** | 0.205 | 6.78 | **6.14** |
| **52** | 19.5 | 4.45 | **23.9** | **4.28** | 0.154 | 0.261 | **0.383** | **0.00949** | 0.0132 | 0.00532 | **0.0179** | 1.40 | 7.51 | **7.99** | 0.0483 | 0.258 | **0.275** |
| **53** | 18.0 | 4.06 | **22.0** | **4.42** | 0.191 | 0.251 | **0.411** | **0.00793** | 0.0130 | 0.00491 | **0.0173** | 1.74 | 7.80 | **8.57** | 0.0583 | 0.331 | **0.349** |
| **54** | 21.5 | 5.00 | **26.5** | **4.30** | 0.204 | 0.276 | **0.446** | **0.00942** | 0.0167 | 0.00626 | **0.0221** | 1.86 | 8.37 | **9.20** | 0.0881 | 0.489 | **0.516** |
| **55** | 14.6 | 3.25 | **17.8** | **4.47** | 0.197 | 0.223 | **0.393** | **0.00664** | 0.0110 | 0.00392 | **0.0144** | 1.72 | 7.42 | **8.23** | 0.0499 | 0.226 | **0.249** |
| **56** | 18.3 | 4.11 | **22.4** | **4.46** | 0.173 | 0.242 | **0.385** | **0.00857** | 0.0134 | 0.00516 | **0.0179** | 1.50 | 7.21 | **7.83** | 0.0706 | 0.419 | **0.439** |
| **Cannabis resin** | | | | | | | | | | | | | | | | | |
| **57** | 28.1 | 8.89 | **37.0** | **3.16** | 0.228 | 0.0151 | **0.241** | **0.963** | 0.0468 | 0.00191 | **0.0485** | 1.11 | 0.612 | **1.65** | 0.0931 | 0.243 | **0.307** |
| **58** | 29.7 | 8.95 | **38.7** | **3.32** | 0.239 | 0.0119 | **0.249** | **0.366** | 0.210 | 0.000219 | **0.212** | 0.670 | 0.649 | **1.28** | 0.877 | 5.46 | **5.67** |
| **59** | 4.06 | 1.33 | **5.39** | **3.06** | 1.30 | 0.142 | **1.43** | **2.08** | 0.391 | 0.0175 | **0.406** | 12.4 | 2.64 | **14.7** | 2.44 | 2.50 | **4.63** |
| **60** | 32.0 | 9.85 | **41.9** | **3.25** | 0.230 | 0.0104 | **0.239** | **0.355** | 0.248 | <LOQ | **0.248** | 0.705 | 0.776 | **1.39** | 1.44 | 10.9 | **11.0** |
| **61** | 11.8 | 2.91 | **14.7** | **4.05** | 1.08 | 1.20 | **2.13** | **0.320** | 0.328 | 0.117 | **0.431** | 0.812 | 0.688 | **1.41** | 6.35 | 3.31 | **9.25** |
| **62** | 0.000782 | 0.000825 | **0.00161** | **0.947** | 0.0119 | nd | **0.0119** | **0.00299** | 0.0110 | 0.000455 | **0.0114** | 67.3 | 0.0490 | **67.3** | 1.49 | 1.75 | **3.46** |
| **63** | 29.0 | 9.01 | **38.0** | **3.22** | 0.216 | <LOQ | **0.216** | **0.189** | 0.0285 | <LOQ | **0.0285** | 0.681 | 0.627 | **1.23** | 1.75 | 11.4 | **11.7** |
| **64** | 28.0 | 8.87 | **36.9** | **3.16** | 0.220 | <LOQ | **0.220** | **0.194** | 0.0283 | <LOQ | **0.0283** | 0.567 | 0.502 | **1.01** | 1.68 | 8.41 | **9.12** |
| **65** | 9.56 | 4.68 | **14.2** | **2.04** | 0.0163 | nd | **0.0163** | **0.0131** | 0.0233 | nd | **0.0233** | 16.7 | 0.603 | **17.2** | 0.219 | 0.0350 | **0.250** |
| **66** | 22.8 | 6.19 | **29.0** | **3.68** | 0.281 | <LOQ | **0.281** | **0.716** | 0.0395 | nd | **0.0935** | 0.0431 | 0.0233 | **0.0635** | 3.72 | 11.3 | **13.7** |
| **67** | 0.0332 | 0.0110 | **0.0442** | **3.00** | 0.0261 | 0.00421 | **0.0298** | **0.00244** | 0.955 | 0.000385 | **0.955** | 0.947 | 0.763 | **1.62** | 2.51 | 10.6 | **11.8** |
| **68** | 24.4 | 6.81 | **31.2** | **3.59** | 0.322 | 0.115 | **0.427** | **0.589** | 0.0856 | 0.0245 | **0.105** | 0.681 | 0.569 | **1.18** | 1.19 | 9.19 | **9.26** |
| **69** | 0.0271 | 0.00830 | **0.0354** | **3.27** | 0.0362 | 0.00761 | **0.0429** | **0.00532** | 0.814 | 0.00187 | **0.815** | 0.886 | 0.786 | **1.58** | 2.41 | 8.66 | **10.0** |
| **Edibles** (gummies) | | | | | | | | | | | | | | | | | |
| **70** | 0.00214 | 0.00435 | **0.00698** | **2.26** | nd | nd | nd | nd | 0.00206 | nd | **0.00206** | <LOQ | nd | <LOQ | nd | nd | **nd** |
| **71** | 0.00651 | 0.00299 | **0.00950** | **2.18** | nd | nd | nd | nd | 0.00242 | nd | **0.00242** | <LOQ | nd | <LOQ | nd | nd | **nd** |
| **72** | 0.0282 | 0.0120 | **0.0401** | **2.35** | <LOQ | nd | <LOQ | **0.000199** | <LOQ | nd | <LOQ | <LOQ | 0.0000660 | **0.0000506** | nd | nd | **nd** |
| **73** | 0.0445 | 0.158 | **0.202** | **3.54** | 0.000657 | nd | **0.000657** | **0.000609** | 0.000601 | nd | **0.000601** | <LOQ | 0.0000572 | **0.0000460** | nd | nd | **nd** |
| ***** | 0.00462 | 0.0232 | **0.278** | **5.03** | 0.000165 | nd | **0.000165** | **0.000234** | 0.00000485 | nd | **0.0000485** | 0.000601 | 0.000233 | **0.000805** | nd | 0.000121 | **0.000106** |
| **Vape liquids** | | | | | | | | | | | | | | | | | |
| **74** | 51.5 | 19.4 | **70.9** | **2.66** | 0.414 | nd | **0.414** | **0.433** | 0.164 | nd | **0.164** | 0.00997 | 0.0721 | **0.0732** | nd | 0.00463 | **0.00406** |
| **75** | 1.41 | 0.731 | **2.14** | **1.93** | 0.000605 | nd | **0.000605** | **0.00312** | 0.000314 | nd | **0.000314** | 0.00253 | nd | **0.00253** | nd | nd | **nd** |
| **Papers** (presumably soaked with vape liquid) | | | | | | | | | | | | | | | | | |
| **76** | positive | positive | **positive** | **2.86** | positive | nd | **positive** | **positive** | positive | nd | **positive** | positive | nd | **positive** | nd | nd | **nd** |
| **77** | positive | positive | **positive** | **2.87** | positive | nd | **positive** | **positive** | positive | nd | **positive** | positive | nd | **positive** | nd | nd | **nd** |
| **78** | positive | positive | **positive** | **2.85** | positive | nd | **positive** | **positive** | positive | nd | **positive** | positive | nd | **positive** | nd | nd | **nd** |
| **79** | positive | positive | **positive** | **2.75** | positive | nd | **positive** | **positive** | positive | nd | **positive** | positive | nd | **positive** | nd | nd | **nd** |

**Table S3 Raw data - Quantification of HHC, Δ^9^-THC, Δ^8^-THC, CBN, CBD and CBG in the seizure collective.** Seizures are sub-divided into groups according to their sample types and numbered. The separately bought H4CBD gummies are marked with (*). Elevated Δ^9^-THC, Δ^8^-THC and CBN-contents are marked (≥ 0.3 - ≤ 0.5 wt-%, ≥ 0.5 - ≤ 1.0 wt-%, ≥ 1.0 wt-%). The dominant cannabinoid of the carrier material is highlighted. In some cases, a mixture of CBD- and CBG-dominant carrier material of CBD- and CBG-dominant is to be considered. This was considered when the CBD exceeded 15% of the total cannabinoid content in CBG-dominant material or when the CBG content was conspicuously high (> 15 % of total cannabinoid content) in CBD-dominant material.

| **#** | **Quantitative data** (wt-%) | | | | | | | **Qualitative data** (ISTD-Relative response ratios, normalised by dilution factor and weightings) | | | | | | | | |
| --- | --- | --- | --- | --- | --- | --- | --- | --- | --- | --- | --- | --- | --- | --- | --- | --- |
|  | (R)-HHC-O | (S)-HHC-O | **HHC-O_tot_** | **(R)/(S)-HHC-O** | **HHC_tot_**  **(HHC +**  **HHC-O)** | **CBN-O** | **CBN_tot_**  **(CBN +**  **CBN-O)** | **H4CBD**  **(R); (S)** | **(R)/(S)-**  **H4CBD** | **HHCP**  **(R); (S)** | **(R)/(S)-**  **HHCP** | **Δ^9^-THCP** | **Δ^8^-THCP** | **HHCP-O**  **(R); (S)** | **THC-O**  **Σ Δ^8^+Δ^9^** | **THCP-O**  **Σ Δ^8^+Δ^9^** |
| **Cannabis flowers** | | | | | | | | | | | | | | | | |
| **1** | 0.192 | 0.248 | **0.440** | **0.774** | **0.688** | **0.0229** | **0.0654** | nd | na | **26.4; 1.66** | **15.9** | nd | nd | **1.10; 0.266** | **51.8** | nd |
| **2** | 0.799 | 0.982 | **1.78** | **0.814** | **3.32** | **0.0840** | **0.125** | nd | na | **109; 15.4** | **7.10** | **1.42** | **0.832** | **0.410; 0.176** | **21.31** | nd |
| **3** | 0.00106 | 0.00125 | **0.00231** | **0.844** | **27.2** | nd | na | nd | na | nd | na | **0.515** | **0.0534** | nd | nd | nd |
| **4** | 0.0393 | 0.0867 | **0.126** | **0.453** | **9.35** | nd | na | nd | na | nd | na | nd | nd | nd | **1.42** | nd |
| **5** | <LOQ | <LOQ | <LOQ | na | na | nd | na | nd | na | nd | na | nd | nd | nd | nd | nd |
| **6** | nd | nd | nd | na | na | nd | na | nd | na | nd | na | nd | nd | nd | nd | nd |
| **7** | 0.00460 | 0.00778 | **0.0124** | **0.592** | **25.4** | **0.000322** | **0.0626** | **1.48; 0.498** | **2.98** | nd | na | nd | nd | nd | nd | nd |
| **8** | 0.00247 | 0.00699 | **0.00946** | **0.353** | **41.5** | <LOQ | na | nd | na | nd | na | nd | nd | nd | nd | nd |
| **9** | <LOQ | <LOQ | nd | na | na | nd | na | nd | na | nd | na | nd | nd | nd | nd | nd |
| **10** | nd | nd | nd | na | na | nd | na | **5.42; 1.71** | **3.17** | **111; 7.95** | **14.0** | **1.44** | **1.21** | **0.640; 0.140** | nd | nd |
| **11** | nd | nd | nd | na | na | nd | na | **4.13; 1.71** | **3.90** | nd | na | nd | nd | nd | nd | nd |
| **12** | nd | nd | nd | na | na | nd | na | **25.7; 7.53** | **3.41** | nd | na | nd | nd | nd | nd | nd |
| **13** | nd | nd | nd | na | na | nd | na | **6.60; 1.46** | **4.52** | **2.04; 0.190** | **10.7** | **1.87** | nd | nd | nd | nd |
| **14** | nd | nd | nd | na | na | nd | na | nd | na | nd | na | nd | nd | nd | nd | nd |
| **15** | 0.00626 | 0.00808 | **0.0143** | **0.775** | **52.3** | nd | na | **13.7; 4.58** | **2.99** | nd | na | nd | nd | nd | **4.69** | nd |
| **16** | nd | nd | nd | na | na | nd | na | **8.55; 2.15** | **3.98** | **2.67; 0.200** | **13.4** | **2.76** | **0.22** | nd | nd | nd |
| **17** | nd | nd | nd | na | na | nd | na | **30.4; 3.82** | **7.96** | nd | na | nd | nd | nd | nd | nd |
| **18** | nd | nd | nd | na | na | nd | na | nd | na | **18.8; 1.99** | **9.45** | **0.209** | **0.0995** | nd | nd | nd |
| **19** | nd | nd | nd | na | na | nd | na | **1.82; 0.584** | **3.12** | **96.3; 7.94** | **12.1** | **1.20** | **0.970** | **0.446; 0.109** | nd | nd |
| **20** | nd | nd | nd | na | na | nd | na | **2.14; 0.734** | **2.92** | **0.793; 0.0569** | **13.9** | **1.05** | **1.05** | **0.693; 0.191** | nd | nd |
| **21** | <LOQ | <LOQ | <LOQ | na | na | nd | na | **5.99; 1.75** | **3.43** | nd | na | nd | nd | nd | nd | nd |
| **22** | nd | nd | nd | na | na | nd | na | **2.27; 0.507** | **4.47** | **60.3; 6.85** | **8.81** | **0.896** | **0.368** | nd | nd | nd |
| **23** | <LOQ | <LOQ | <LOQ | na | na | nd | na | **4.45; 1.48** | **3.01** | nd | na | nd | nd | nd | nd | nd |
| **24** | nd | nd | nd | na | na | nd | na | nd | na | nd | na | nd | nd | nd | nd | nd |
| **25** | nd | nd | nd | na | na | nd | na | **3.37**; nd | na | nd | na | nd | nd | nd | nd | nd |
| **26** | <LOQ | 0.00512 | **0.00512** | na | **12.4** | nd | na | nd | na | nd | na | nd | nd | nd | nd | nd |
| **27** | nd | nd | nd | na | na | nd | na | **61.7; 25.4** | **2.43** | **68.9; 5.74** | **12.0** | **1.29** | **0.310** | **0.667; 0.197** | nd | nd |
| **28** | nd | nd | nd | na | na | nd | na | **225; 45.0** | **5.00** | nd | na | **229** | **5.45** | nd | nd | nd |
| **29** | nd | nd | nd | na | na | nd | na | **1.29; 0.353** | **3.67** | nd | na | **0.667** | nd | nd | nd | nd |
| **30** | <LOQ | <LOQ | <LOQ | na | na | nd | na | nd | na | nd | na | nd | nd | nd | nd | nd |
| **31** | 0.00226 | 0.00445 | **0.00671** | **0.0509** | **11.2** | nd | na | nd | na | nd | na | nd | nd | nd | nd | nd |
| **32** | <LOQ | <LOQ | <LOQ | na | na | nd | na | nd | na | nd | na | nd | nd | nd | nd | nd |
| **33** | nd | nd | nd | na | na | nd | na | nd | na | **8.01; 2.85** | **2.81** | **0.477** | **0.375** | nd | nd | nd |
| **34** | nd | nd | nd | na | na | nd | na | **202; 89.8** | **2.25** | **175; 13.3** | **13.2** | **15.5** | **0.871** | **1.03; 0.0980** | nd | nd |
| **35** | nd | nd | nd | na | na | nd | na | **0.739; 0.202** | **3.65** | **7.98;** nd | na | **40.9** | **98.1** | **2870; 1120** | nd | **2.04** |
| **36** | nd | nd | nd | na | na | nd | na | **0.636; 0.170** | **3.74** | **7.21; 0.285** | **25.3** | **32.4** | **81.0** | **2270; 872** | nd | **1.76** |
| **37** | nd | nd | nd | na | na | nd | na | **0.481; 0.134** | **3.59** | **6.35; 0.327** | **19.4** | **25.7** | **93.9** | **1730; 741** | nd | **1.46** |
| **38** | nd | nd | nd | na | na | nd | na | **86.9; 36.3** | **2.40** | **115; 8.85** | **13.0** | **4.25** | **0.365** | **1.46; 0.242** | nd | nd |
| **39** | nd | nd | nd | na | na | nd | na | **0.924; 0.249** | **3.72** | **9.39; 0.438** | **21.4** | **49.0** | **112** | **2820; 1350** | nd | **2.40** |
| **40** | nd | nd | nd | na | na | nd | na | **0.547; 0.146** | **3.76** | **6.02; 0.283** | **21.3** | **29.0** | **70.9** | **2020; 771** | nd | **1.54** |
| **41** | nd | nd | nd | na | na | nd | na | **75.0; 34.1** | **2.20** | **65.0; 4.61** | **14.1** | **4.44** | **0.356** | **1.19; 0.226** | nd | nd |
| **42** | nd | nd | nd | na | na | nd | na | **0.799; 0.219** | **3.66** | **8.28; 0.351** | **23.6** | **47.3** | **102** | **2770; 1350** | nd | **2.18** |
| **43** | nd | nd | nd | na | na | nd | na | **97.7; 41.4** | **2.36** | **106; 9.80** | **10.8** | **5.04** | **0.492** | **1.76; 0.359** | nd | nd |
| **#** | (R)-HHC-O | (S)-HHC-O | **HHC-O_tot_** | **(R)/(S)-HHC-O** | **HHC_tot_**  **(HHC +**  **HHC-O)** | **CBN-O** | **CBN_tot_**  **(CBN +**  **CBN-O)** | **H4CBD**  **(R); (S)** | **(R)/(S)-**  **H4CBD** | **HHCP**  **(R); (S)** | **(R)/(S)-**  **HHCP** | **Δ^9^-THCP** | **Δ^8^-THCP** | **HHCP-O**  **(R); (S)** | **THC-O**  **Σ Δ^8^+Δ^9^** | **THCP-O**  **Σ Δ^8^+Δ^9^** |
| **Cannabis flowers** (continued) | | | | | | | | | | | | | | | | |
| **44** | nd | nd | nd | na | na | nd | na | **135; 61.1** | **2.21** | **131; 10.3** | **12.7** | **10.2** | **0.643** | **1.23; 0.189** | nd | nd |
| **45** | 0.000598 | 0.000495 | **0.00109** | **1.21** | **9.61** | nd | na | nd | na | **0.560; 0.188** | **2.98** | nd | nd | **0.188; 0.0847** | nd | nd |
| **46** | nd | nd | nd | na | na | nd | na | **52.4; 24.1** | **2.18** | **54.6; 4.41** | **12.4** | **0.535** | **0.249** | **1.66; 0.493** | nd | nd |
| **47** | nd | nd | nd | na | na | nd | na | **0.417; 0.112** | **3.71** | **5.11; 0.203** | **25.2** | **25.0** | **56.9** | **1990; 764** | nd | **1.42** |
| **48** | <LOQ | <LOQ | <LOQ | na | na | nd | na | nd | na | nd | na | nd | nd | nd | nd | nd |
| **49** | nd | nd | nd | na | na | nd | na | nd | na | nd | na | **183** | **52.0** | **2.25; 0.873** | nd | nd |
| **50** | <LOQ | <LOQ | <LOQ | na | na | nd | na | nd | na | nd | na | nd | nd | nd | nd | nd |
| **51** | nd | nd | nd | na | na | nd | na | **110; 49.0** | **2.24** | **106; 8.34** | **12.7** | **1.34** | **0.401** | **1.25; 0.372** | nd | nd |
| **52** | <LOQ | <LOQ | <LOQ | na | na | nd | na | nd | na | nd | na | **1.45** | **0.409** | nd | nd | nd |
| **53** | <LOQ | <LOQ | <LOQ | na | na | nd | na | nd | na | nd | na | **3.21** | **0.995** | nd | nd | nd |
| **54** | <LOQ | <LOQ | <LOQ | na | na | nd | na | nd | na | nd | na | **7.90** | **2.16** | nd | nd | nd |
| **55** | <LOQ | <LOQ | <LOQ | na | na | nd | na | nd | na | nd | na | **0.427** | **0.122** | nd | nd | nd |
| **56** | <LOQ | <LOQ | <LOQ | na | na | nd | na | nd | na | nd | na | **6.71** | **1.79** | nd | nd | nd |
| **Cannabis resin** | | | | | | | | | | | | | | | | |
| **57** | 0.00284 | 0.00744 | **0.0103** | **0.381** | **36.9** | nd | na | **0.311; 0.0823** | **3.78** | **0.137; 0.0390** | **3.51** | nd | nd | nd | **0.468** | nd |
| **58** | 0.0122 | 0.0319 | **0.0441** | **0.382** | **38.7** | <LOQ | na | **0.307; 0.0801** | **3.83** | **0.144; 0.0317** | **4.52** | nd | nd | nd | **0.318** | nd |
| **59** | nd | nd | nd | na | na | nd | na | nd | na | **34.5; 9.18** | **3.76** | **1.72** | **0.546** | nd | **0.459** | nd |
| **60** | 0.0120 | 0.0283 | **0.0404** | **0.424** | **41.9** | nd | na | nd | na | nd | na | nd | nd | nd | nd | nd |
| **61** | 0.00192 | 0.00297 | **0.00487** | **0.645** | **14.7** | nd | na | **0.164; 0.0738** | **2.22** | **0.456**; nd | na | **4.75** | **0.138** | nd | **0.482** | nd |
| **62** | nd | nd | nd | na | na | nd | na | nd | na | **136; 18.7** | **7.29** | **1.16** | **0.259** | **0.523; 0.209** | nd | nd |
| **63** | 0.00281 | 0.00426 | **0.00707** | **0.659** | **14.3** | nd | na | **6.55; 2.13** | **3.07** | nd | na | nd | nd | nd | nd | nd |
| **64** | 0.00296 | 0.00445 | **0.00742** | **0.665** | **36.9** | nd | na | **7.00; 2.01** | **3.48** | nd | na | nd | nd | nd | nd | nd |
| **65** | 0.00331 | 0.00487 | **0.00818** | **0.680** | **14.3** | nd | na | nd | na | nd | na | nd | nd | nd | nd | nd |
| **66** | 0.000952 | 0.00144 | **0.00239** | **0.662** | **29.0** | nd | na | **0.219; 0.0581** | **3.77** | **0.193; 0.0291** | **6.64** | nd | nd | **0.383; 0.124** | nd | nd |
| **67** | nd | nd | nd | na | na | nd | na | **2290; 663** | **3.45** | nd | na | **251** | **35.3** | **0.178; 0.0507** | nd | nd |
| **68** | 0.00141 | 0.00220 | **0.00361** | **0.640** | **31.2** | nd | na | **0.180; 0.0737** | **2.45** | **0.203; 0.0356** | **5.71** | nd | nd | **0.145; 0.0610** | nd | nd |
| **69** | nd | nd | nd | na | na | nd | na | **2410; 677** | **3.55** | nd | na | **27.4** | **172** | nd | nd | nd |
| **Edibles** (gummies) | | | | | | | | | | | | | | | | |
| **70** | 0.143 | 0.311 | **0.453** | **0.460** | **0.407** | **0.0439** | **0.0407** | nd | na | nd | na | nd | nd | **0.0415; 0.0356** | **0.109** | nd |
| **71** | 0.243 | 0.531 | **0.773** | **0.457** | **0.692** | **0.0708** | **0.0647** | nd | na | nd | na | nd | nd | **0.0701; 0.0616** | **0.170** | nd |
| **72** | 0.254 | 0.222 | **0.476** | **1.14** | **0.460** | **0.000736** | **0.000648** | nd | na | nd | na | nd | nd | **0.0523; 0.0248** | **1.33** | nd |
| **73** | 0.0000630 | 0.0000818 | **0.000145** | **0.770** | **0.202** | nd | na | nd | na | nd | na | nd | nd | nd | nd | nd |
| ***** | <LOQ | <LOQ | <LOQ | na | na | nd | na | nd | na | nd | na | nd | nd | nd | nd | nd |
| **Vape liquids** | | | | | | | | | | | | | | | | |
| **74** | 0.0343 | 0.0354 | **0.0697** | **0.971** | **70.9** | <LOQ | na | **31.1; 11.2** | **2.78** | **1.43; 0.264** | **5.42** | nd | nd | nd | nd | nd |
| **75** | <LOQ | <LOQ | <LOQ | na | na | nd | na | nd | na | **0.00482; 0.00195** | **2.47** | nd | nd | nd | nd | nd |
| **Papers** (presumably soaked with vape liquid) | | | | | | | | | | | | | | | | |
| **76** | <LOQ | <LOQ | <LOQ | na | na | nd | na | nd | na | nd | na | nd | nd | nd | nd | nd |
| **77** | <LOQ | <LOQ | <LOQ | na | na | nd | na | nd | na | nd | na | nd | nd | nd | nd | nd |
| **78** | <LOQ | <LOQ | <LOQ | na | na | nd | na | nd | na | nd | na | nd | nd | nd | nd | nd |
| **79** | <LOQ | <LOQ | <LOQ | na | na | nd | na | nd | na | nd | na | nd | nd | nd | nd | nd |

**Table S4 Raw data - Quantification of HHC-O and CBN-O as well as qualitative analysis of further derivatives in the seizure collective.** Elevated HHC-O (> 0.1 wt-%) and HHCP-O contents are marked. Isolated detections of HHCP-O without HHC-O or HHC-P are highlighted.
